# Repeatability of and Relationship between Potential COPD Biomarkers in Bronchoalveolar Lavage, Bronchial Biopsies, Serum, and Induced Sputum

**DOI:** 10.1371/journal.pone.0046207

**Published:** 2012-10-04

**Authors:** Stefan Röpcke, Olaf Holz, Gereon Lauer, Meike Müller, Susanne Rittinghausen, Peter Ernst, Gezim Lahu, Martin Elmlinger, Norbert Krug, Jens M. Hohlfeld

**Affiliations:** 1 Department of Biomarker Development, Nycomed GmbH, Konstanz, Germany; 2 Department of Clinical Airway Research, Fraunhofer Institute for Toxicology and Experimental Medicine (ITEM), Hannover, Germany; 3 Biomedical Research in Endstage and Obstructive Lung Disease Hannover (BREATH), Member of the German Center for Lung Research, Hannover, Germany; 4 Department of Toxicology and Environmental Hygiene, Fraunhofer Institute for Toxicology and Experimental Medicine (ITEM), Hannover, Germany; 5 GeneData AG, Basel, Switzerland; University of Tübingen, Germany

## Abstract

Chronic Obstructive Pulmonary Disease (COPD) is a chronic inflammatory disease, primarily affecting the airways. Stable biomarkers characterizing the inflammatory phenotype of the disease, relevant for disease activity and suited to predict disease progression are needed to monitor the efficacy and safety of drug interventions. We therefore analyzed a large panel of markers in bronchoalveolar lavage, bronchial biopsies, serum and induced sputum of 23 healthy smokers and 24 smoking COPD patients (GOLD II) matched for age and gender. Sample collection was performed twice within a period of 6 weeks. Assays for over 100 different markers were validated for the respective matrices prior to analysis. In our study, we found 51 markers with a sufficient repeatability (intraclass correlation coefficient >0.6), most of these in serum. Differences between groups were observed for markers from all compartments, which extends (von-Willebrand-factor) and confirms (e.g. C-reactive-protein, interleukin-6) previous findings. No correlations between lung and serum markers were observed, including A1AT. Airway inflammation defined by sputum neutrophils showed only a moderate repeatability. This could be improved, when a combination of neutrophils and four sputum fluid phase markers was used to define the inflammatory phenotype.In summary, our study provides comprehensive information on the repeatability and interrelationship of pulmonary and systemic COPD-related markers. These results are relevant for ongoing large clinical trials and future COPD research. While serum markers can discriminate between smokers with and without COPD, they do not seem to sufficiently reflect the disease-associated inflammatory processes within the airways.

## Introduction

COPD is characterized by chronic airway inflammation, dyspnea, reduced exercise tolerance, cough, increased mucus production, and can lead to emphysema [1], [2]. Structural and functional abnormalities of the bronchial vasculature have been associated with the development of COPD [3], [4]. COPD is a complex multi-organ disease, but the diagnosis relies predominantly on patient-reported symptoms and spirometry [5], which have limitations in terms of accuracy, specificity and sensitivity [1]. Currently there are no reliable, validated, and easily accessible biomarkers that reflect the inflammatory state of the airways [6]–[8]. In addition, little is known about factors that define disease activity or progression [9], making COPD an active area of clinical and pharmacological research, with national (COSYCONET) [10] and international (ECLIPSE) [11] large cohort trials in progress.

These trials and our efforts in this study reflect the need for biomarkers that enable researchers and physicians to adequately measure airway inflammation in COPD and to perform a more precise diagnosis of disease states in clinical practice, which could lead to earlier recognition of exacerbations and more tailored interventions. Inflammatory biomarkers could serve as early signals for efficacy or adverse reactions during investigational interventions and would advance pharmacological and clinical research.

Increased numbers and altered activities of pulmonary inflammatory cells as well as enhanced elastolysis are a common feature of COPD. Factors like neutrophil elastase (NE) or matrix metalloproteases (MMP) in bronchoalveolar lavage (BAL) or sputum, are considered as markers for degradation and repair processes [5]. Among the best-studied systemic markers in COPD are the acute phase protein CRP (C-reactive protein) and fibrinogen, which was recently shown to be the most repeatable marker in a large panel study of serum markers analyzed in the ECLIPSE study [12]. Serum CRP is associated with mortality, morbidity, number of exacerbations, and inversely related to lung function indices [13], [14]. Systemic inflammation is also reflected by increased serum concentrations of IL-6, TNFα and MCP-1 in COPD patients [1], [15]. Serum IL-6 and CRP moderately correlate, are fairly stable over 1 year [16] and are increased in COPD patients with metabolic syndrome [17]. However, the extent to which serum markers mirror ongoing inflammatory processes within the lung is largely unknown. Recently published data from the ECLIPSE study showed only a weak association between sputum neutrophils and 4 serum markers [18]. While there is a lot of data available on potential biomarkers for COPD, only a few studies have addressed the issue of repeatability of multiple systemic and pulmonary markers [12], [16], [18], [19].

In our study, we therefore assessed the repeatability of a broad panel of markers from serum, sputum, BAL and bronchial biopsies, by collecting samples twice within 6 weeks. Prior to this study all assays for the analysis of biomarkers were extensively validated with samples from the respective matrices. In contrast to other studies, we focused on disease-related differences and aimed to avoid a bias due to active smoking by comparing age and gender matched active smokers with and without COPD (GOLD II). In addition, we compared markers not only between groups but also between the different sampling sites, especially to investigate to what extent serum markers relate to inflammatory markers within the airways.

## Results

### Patient demographics

Table 1 lists the demographics of the study groups. Subjects were matched with respect to gender and age. All were current smokers, verified by urine cotinine measurements (mean within patient variation coefficient: 0.36). COPD patients (GOLD II) reported slightly higher daily cigarette consumption, but no significant differences in cotinine levels between groups were observed. COPD patients had lower lung function values, oxygen saturation as well as a lower peak exercise capacity at screening.

**Table 1:** Demographic and physiological parameters.

	Healthy Smokers (N = 23)	COPD Smokers (N = 24)
female/male	6/17	6/18
age [years]	54 (42, 65)	54 (46, 68)
height [cm]	176.4±11.3	174.7±7.4
weight [kg]	79.6±14.0	77.4±12.1
BMI [kg/m^2^]	25.4±2.5	25.3±3.4
Pack-years	39±23.2	49.2±12.6***
Cig. per day	20.7±8.9	25.5±6.9***
Cotinine (ng/mL): Scr.	1262±722	1561±968
V1	1378±722	1772±1107
V3	1451±823	1779±1012
FEV_1_ [L]	3.8±0.8	2.0±0.3***
FEV_1_ % pred.	112.5±14.1	60.5±6.8***
FVC [L]	5.1±1.0	4.2±0.9**
FEV_1_/FVC [%]	75.4±5.0	48.7±7.4***
pO2 [mm Hg]	82.9±9.7	73.3±5.3***
WPeak [W]	154.8±35.1	110.8±3.4***

Values are presented as mean ± SD, except for age where we report median (Min, Max);

* : p<0.05,

** : p<0.01,

*** : p<0.001 (Scr. = Screening).

### Pre-Validation of Immunoassays

Before measurement of study samples, all immunoassays were validated for blood, sputum and BAL fluid using pooled samples from at least 6 independent donors. In total, 107 different assays were tested. The samples were spiked and tested for accuracy, recovery and linearity. The optimal dilution of samples for all analytes and all compartments are listed in the online supplement (Table S1). Further validation details are listed under Material and Methods.

### Repeatability of biomarkers

**Figure 1:**
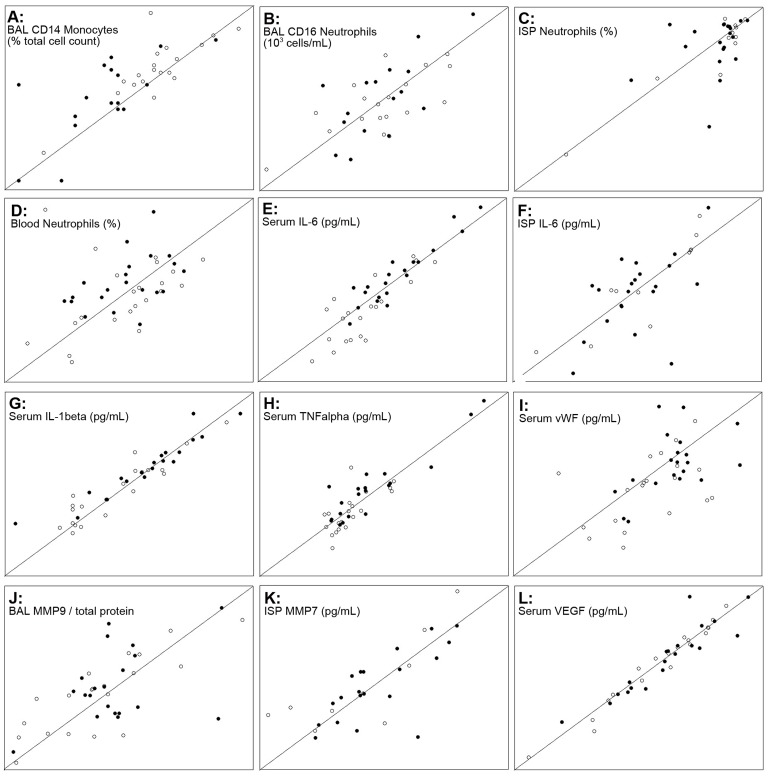
Selected correlations between visits. Correlation between samples collected in 2 visits within a time period of up to 6 weeks. The figure shows selected cellular biomarkers (A–D) and pro-inflammatory cytokines (E–H) from serum, BAL and ISP and examples for proteases (J, K), a glycoprotein and a growth-factor (I, L). The line of identity is displayed in all individual graphs. Data is displayed on log scales. The range of concentrations for each selected marker can be found in table 4 and in the tables of the online supplement. Filled symbols: COPD smokers, open symbols: healthy smokers.

Tables 2 and 3 list all markers with intraclass correlation coefficients (ICC) >0.6 for all subjects and for both groups separately. All correlations were checked for potential bias due to outliers and selected correlations between visits are displayed in Figure 1. Overall, markers in serum showed the best repeatability. While the ICC values in the lung compartments were generally higher in healthy smokers, serum markers appeared most often to be more robust in COPD patients. Sputum macrophages and MMP9 were repeatable only in the COPD patients, while sputum neutrophils were more stable in healthy smokers. In BAL, cellular markers and high abundant proteins, such as albumin (HSA) and alpha-1-antitrypsin (A1AT) were repeatable. Other mediators had to be normalized to total protein to reach ICC values >0.6.

**Table 2:** List of repeatable biomarkers in the lung compartments (for all markers with ICC>0.60).

ANALYTE	ALL		COPD smokers			Healthy smokers		
	ICC	r	ICC	r	SD	ICC	r	SD
**BAL**								
CD14 Mono	0.77	0.70	0.68	0.68	2.5	0.80	0.81	2.1
Calprotectin/TP	0.73	0.75	0.46	0.54	1.7	0.83	0.83	2.3
HSA	0.69	0.72	0.50	0.51	1.5	0.73	0.80	1.6
CD16 NG	0.67	0.66	0.67	0.68	3.5	0.69	0.68	3.2
TCC	0.65	0.65	0.61	0.60	2.4	0.71	0.72	2.1
IL-8/TP	0.65	0.64	0.58	0.57	2.3	0.63	0.63	2.3
MMP-9/TP	0.65	0.65	0.43	0.41	3.3	0.81	0.81	4.0
total-protein	0.64	0.66	0.60	0.65	1.6	0.69	0.68	1.6
NELA/TP	0.62	0.62	0.43	0.43	2.8	0.82	0.84	2.9
a1-Antitrypsin	0.61	0.65	0.52	0.62	1.9	0.63	0.63	1.9
MPO/TP	0.39	0.41	0.04	0.04	2.5	**0.65**	0.76	2.8
**SPUTUM**								
MMP 7	0.76	0.75	0.73	0.72	2.7	0.82	0.82	4.1
EP (%)	0.76	0.76	0.82	0.82	3.3	0.64	0.62	3.2
IL-6	0.69	0.71	0.52	0.54	2.5	0.89	0.89	3.4
HSA	0.61	0.60	0.57	0.56	2.2	0.58	0.61	2.2
NG (%)	0.59	0.55	0.33	0.31	1.8	**0.87**	0.85	2.1
a1-Antitrypsin	0.56	0.55	0.36	0.35	1.6	**0.72**	0.70	1.7
AM	0.53	0.52	**0.66**	0.66	2.2	0.39	0.38	2.5
TIMP-1	0.52	0.51	0.33	0.37	1.6	**0.70**	0.68	2.4
MMP-1	0.37	0.40	0.24	0.27	2.6	**0.62**	0.63	2.7
MMP-9/TP	0.24	0.32	**0.68**	0.72	2.5	−0.03	0.06	4.0

Intraclass correlation coefficients (ICC) were derived from one-way ANOVA tables as the ratio of variance among subjects to total variance based on 2 measurements over a 6 week period (for log transformed data only) r: Pearson correlation coefficient. Data is sorted by matrix and decreasing ICC as derived from all subjects. Some markers are listed due to ICC>0.6 in the subgroups (in bold). Mean SD (Standard Deviation) values were derived from log-transformed data of the 2 visits, transformed again and listed for the 2 subgroups. This way these values are factors. To derive the SD value, that together with the median values of a marker (listed in table 4 and in the Online Supplement) can be used for sample size and power calculations, the respective median needs to be multiplied and divided by the value listed above (Example for HSA in BAL fluid of COPD patients: Approximate level of HSA in BAL is 11 µg/mL (table 4), the SD is 1.5×11–11/1.5 = 16.5–7.3 = 9.2 µg/mL). CD14 monocytes are displayed as % total cell count, CD16 neutrophils are displayed as 10^3^cells/mL, TP: normalized to total protein, Mono: monocytes, NG: neutrophils, TCC: total cell count, EP: non-squamous epithelia cells, AM: macrophages.

**Table 3:** List of repeatable systemic biomarkers (for all markers with ICC>0.60).

ANALYTE	ALL		COPD smokers			Healthy smokers		
	ICC	r	ICC	r	SD	ICC	r	SD
**SERUM**								
Leptin	0.97	0.97	0.97	0.97	2.9	0.96	0.96	3.8
VEGF	0.95	0.95	0.91	0.91	1.8	0.98	0.98	2.2
CREATININE	0.94	0.94	0.96	0.96	1.3	0.90	0.90	1.2
IL-1beta	0.93	0.94	0.92	0.95	2.8	0.92	0.92	2.8
IGFBP-2	0.90	0.90	0.88	0.88	1.8	0.92	0.92	1.7
MIP-1alpha	0.88	0.89	0.92	0.91	1.7	0.72	0.72	1.3
IL-2	0.88	0.89	0.89	0.91	3.3	0.85	0.86	3.0
TNF-alpha	0.88	0.88	0.92	0.92	2.1	0.70	0.72	1.5
IL-6	0.88	0.90	0.93	0.93	2.6	0.77	0.82	2.6
MIP-1beta	0.85	0.87	0.86	0.86	1.6	0.82	0.88	1.4
IL-15	0.85	0.85	0.87	0.88	2.0	0.72	0.73	1.5
IFN-alpha	0.84	0.85	0.91	0.92	1.7	0.58	0.56	1.4
IL-12p40/p70	0.83	0.86	0.85	0.89	1.5	0.76	0.78	1.2
MMP-1	0.83	0.83	0.82	0.81	2.2	0.84	0.84	2.1
IL-7	0.82	0.82	0.80	0.79	1.6	0.79	0.80	1.5
IFN-gamma	0.82	0.83	0.86	0.87	1.9	0.70	0.73	1.6
IGF-II	0.80	0.82	0.82	0.86	1.3	0.76	0.76	1.2
IGF-I	0.77	0.76	0.79	0.80	1.2	0.74	0.73	1.2
CRP	0.76	0.76	0.77	0.79	2.0	0.67	0.65	2.7
Serotonin	0.75	0.79	0.74	0.84	1.3	0.77	0.78	1.3
PDGF-AA	0.72	0.82	0.65	0.82	1.4	0.79	0.83	1.4
IL-8	0.72	0.81	0.78	0.80	2.0	0.59	0.86	1.6
Calprotectin	0.72	0.72	0.72	0.71	1.9	0.72	0.72	1.9
NELA	0.72	0.72	0.76	0.76	2.0	0.65	0.65	1.8
IGFBP-1	0.71	0.74	0.71	0.74	2.5	0.72	0.73	2.2
Eotaxin	0.70	0.69	0.68	0.68	1.5	0.73	0.80	1.4
HGF	0.69	0.71	0.83	0.87	1.6	0.47	0.46	1.6
MIG	0.69	0.74	0.68	0.72	1.6	0.67	0.72	1.5
IL-2R	0.64	0.74	0.66	0.78	1.4	0.61	0.72	1.4
LBP	0.63	0.70	0.77	0.85	1.3	0.47	0.52	1.3
TGF-beta	0.62	0.67	0.69	0.77	1.4	0.57	0.63	1.4
PDGF-AB/BB	0.62	0.70	0.67	0.74	1.6	0.50	0.61	1.3
HSA	0.60	0.59	0.74	0.73	1.1	0.32	0.31	1.1
MMP-9	0.49	0.50	0.24	0.24	1.4	**0.63**	0.74	1.5
**URINE**								
CREATININE	0.77	0.51	0.79	0.47	2.4	0.53	0.57	1.6

Intraclass (ICC) and Pearsons (r) correlation coefficients for markers in serum and urine (see Legend table 2 for further information).

### Differences between groups

Table 4 lists the markers that were significantly different between COPD patients and healthy smokers and shows the values for both visits. Some markers showed differences only in the male or in the female subjects. To address potential dilution effects, the fluid phase markers in sputum and BAL were analyzed after normalization to the level of total protein. The data of all markers can be found in the online supplement (Table S2, S3, S4, S5, S6, S7, S8).

**Table 4:** Markers with significant differences between groups.

Analyte	Sample matrix	M	Unit	First visit		Second visit		LME-ANOVA
				healthy smokers	COPD smokers	healthy smokers	COPD smokers	p-value
TCC	BAL		10^6^/mL	0.2 (0.2–0.4)	0.2 (0.1–0.3)	0.2 (0.1–0.3)	0.2 (0.1–0.3)	m: 0.008. f:0.0418
CD14+ MONO	BAL	F	% TC	1.5 (1.2–2.5)	1.1 (0.6–1.6)	1.8 (1.3–2.5)	0.9 (0.5–1.0)	m: 0.0001. f:0.61
CD14+ MONO	BAL	F	10^3^/mL	2.7 (1.6–7.4)	2.4 (0.4–5.8)	3.9 (2.6–6.0)	1.5 (0.5–3.8)	m: 0.00045. f:0.11
a1-Antitrypsin	BAL	E	ng/ml	795 (531–1022)	512 (328–724)	650 (358–1074)	345 (275–480)	m: 0.004. f:0.95
EGF-R	BAL	E	pg/ml	67.3 (53.4–90.3)	56.7 (35.4–72.1)	82.6 (61.6–100.0)	55.5 (40.2–87.4)	m: 0.001.f:0.55
HSA	BAL	E	µg/ml	16.8 (12.5–23.9)	11.7 (7.9–12.9)	17.8 (13.0–22.4)	10.5 (9.2–15.2)	m: 1.12e-05. f:0.44
TIMP-1	BAL	E	ng/ml	2.4 (1.8–3.3)	3.2 (2.2–4.7)	2.7 (1.8–3.3)	4.8 (2.4–8.5)	0.016
a1-Antitrypsin	BAL/TP	E	pg/µg	9.9 (8.1–11.2)	7.7 (5.7–10.6)	8.5 (6.6–11.7)	6.1 (4.4–7.5)	0.004
Calprotectin	BAL/TP	E	ng/µg	0.7 (0.4–1.4)	1.2 (0.8–1.5)	0.7 (0.4–1.1)	0.9 (0.7–1.1)	m: 0.016. f:0.59
EGF-R	BAL/TP	E	pg/µg	1.0 (0.8–1.2)	0.9 (0.6–1.0)	1.0 (0.8–1.2)	0.8 (0.7–1.0)	0.016
HSA	BAL/TP	E	ng/µg	245 (226–268)	185 (169–208)	253 (184–283)	183 (140–215)	1.62E-05
IL-8	BAL/TP	Lu	pg/µg	0.3 (0.2–0.4)	0.4 (0.3–0.8)	0.2 (0.2–0.4)	0.5 (0.3–0.7)	0.025
TIMP-1	BAL/TP	E	ng/µg	0.0 (0.0–0.0)	0.1 (0.0–0.1)	0.0 (0.0–0.0)	0.1 (0.0–0.1)	0.000
ANISOCYTOSIS	blood	H	%	44.2 (42.4–46.8)	46.6 (44.9–47.7)	45.9 (44.1–46.8)	46.8 (45.7–47.7)	0.014
CREATINE KIN.	blood	Ch	U/L	125.0 (96.5–173.0)	89.5 (67.8–126.3)	124.0 (91.0–179.0)	83.0 (65.0–95.0)	0.007
MCV	blood	H	FL	89.9 (88.2–90.9)	94.0 (91.2–95.2)	90.0 (88.6–91.5)	93.5 (91.0–96.7)	0.008
a1-Antitrypsin	serum	E	µg/ml	1.39 (1.31–1.49)	1.47 (1.33–1.70)	1.90 (1.27–2.12)	2.27 (1.57–2.43)	m: 0.014. f:0.57
CRP	serum	Lu	ng/ml	301 (146–474)	540 (368–1018)	232 (110–569)	823 (373–1047)	0.000
HGF	serum	Lu	pg/ml	317 (244–391)	419 (300–568)	311 (217–407)	414 (322–501)	0.022
IL-6	serum	Lu	pg/ml	6.9 (4.0–12.0)	12.7 (7.9–23.3)	5.0 (2.0–10.1)	15.8 (9.0–30.0)	0.002
LTB4	serum	E	µg/ml	1.23 (1.10–1.35)	1.26 (1.06–1.58)	1.21 (1.12–1.36)	1.40 (1.29–1.66)	m: 0.0051. f:0.72
vWF	serum	E	mU/ml	1586 (1248–2077)	2089 (1838–2296)	1523 (984–1778)	1860 (1562–2301)	0.003
a1-Antitrypsin	ISP	E	ng/ml	992 (630–1173)	568 (363–716)	625 (453–1014)	540 (432–693)	0.008
HSA	ISP	E	µg/ml	34.1 (26.9–44.5)	13.1 (8.9–25.4)	27.6 (17.6–40.2)	17.7 (7.6–23.4)	m: 0.0016. f:0.57
MMP 3	ISP	Lu	pg/ml	28.0 (15.7–42.7)	22.4 (11.8–33.2)	40.6 (21.6–56.5)	22.9 (14.5–42.7)	m: 0.009. f:0.29
a1-Antitrypsin	ISP/TP	E	ng/µg	2.4 (2.1–3.1)	1.6 (1.3–2.1)	2.1 (1.6–2.6)	1.5 (1.3–1.9)	m: 0.0006. f:0.728
HSA	ISP/TP	E	ng/µg	77.3 (66.0–99.8)	50.1 (32.9–70.2)	77.0 (56.8–89.4)	54.7 (27.0–65.5)	m: 0.001. f:0.50
CREATININE	urine	EP	mg/dl	160 (125–206)	140 (96–230)	189 (130–272)	128 (48–190)	m: 0.42. f:0.002

Data presented as median (IQR). LME-ANOVA p-value: COPD smokers vs. healthy smokers. M = Method of analysis, TP = normalized to total protein, BAL = bronchoalveolar lavage, ISP = induced sputum, F = Flow cytometry, E = ELISA, Lu = Luminex, H = Hematology, Ch = blood chemistry, EP = Laboratory Eipper Besenthal, Tübingen, Germany.

A difference in total cell numbers and monocytes in BAL was observed in male subjects only. COPD patients had lower BAL concentrations of A1AT, EGF-R, and HSA, but elevated levels of TIMP1, as well as of IL-8 and Calprotectin, two markers associated with neutrophilic airway inflammation. These differences were also seen without normalization to total protein.

Lower creatine kinase concentrations were measured in blood of COPD smokers, while their serum levels of inflammatory mediators, including CRP and IL-6, were higher compared to healthy smokers. In serum of COPD smokers, there were increased levels of von-Willebrandt-factor (vWF), a glycoprotein that is involved in arterial thrombus formation. However there was no significantly negative relationship to partial thromboplastin time (PTT) (r = −0.2), which was clearly visible in healthy smokers (r = −0.75, p<0.0001, Figure S1). This was also observed when comparing the respective data for the individual visits. PTT itself was not significantly reduced in COPD patients and remained in the normal range below 39 seconds. Higher urine cotinine levels tended to be related to serum vWF only in healthy smokers, further supporting the view that smoking-independent factors were responsible for the increased levels of vWF in serum of COPD patients.

In line with BAL, but in contrast to serum, we detected higher A1AT concentrations in induced sputum of healthy smokers. In these subjects, we also found increased numbers of monocytes, as well as higher concentrations of HSA and MMP3.

### Relationship between biomarkers

First, we tested the relationship between those analyte levels that were assessed in more than one matrix to determine the extent to which the concentration of a specific marker in a more easily accessible sample like serum or sputum agrees with its concentration in a matrix that can only be obtained invasively. This was done for each of the two visits separately and, for those markers with sufficient repeatability (see tables 2 and 3), the mean values of the two visits were also used. The analysis was performed for the entire study population, as well as for healthy smokers and COPD smokers separately. Between the two lung compartments (BAL and ISP), correlations for HSA (mean of visits: r = 0.45, p = 0.006), for MMP9 (mean of visits normalized to total protein: r = 0.70, p<0.001), and for the ratio MMP9/TIMP1 (mean of visits: r = 0.53, p = 0.001) were found. While the correlation for MMP9 was more pronounced in healthy smokers (r = 0.91, p<0.001), a closer relationship between central (ISP) and peripheral (BAL) lung was found for the ratio MMP9/TIMP1 in smoking COPD patients (r = 0.75, p<0.001). The best correlation between serum and BAL was detected for Calprotectin in healthy smokers (in BAL normalized to total protein, visit 1: r = 0.54, p = 0.009; visit 2: r = 0.49, p = 0.03). This relationship was not seen in COPD patients. Weak or no correlations were observed for the total cell count and the proportion of individual cells between blood, ISP and BAL, the moderate correlation between the number of neutrophils in bronchial biopsies with the percentage of CD16 positive neutrophils in BAL (r = 0.68, p<0.001, Figure S2) being the exception.

Next, we used an exploratory factor analysis to search for overall relationships between markers in order to test whether any analytes in easily accessible serum samples relates to markers in BAL, biopsies or sputum. A factor analysis was used to structure our large dataset and to reduce it to 3 groups of highly correlated variables (factors). Analysis of complete cellular and biochemical parameters of all sample matrices (log mean values of the two visits) showed that markers associated with neutrophilic inflammation in sputum and BAL (e.g. MMP9, Elastase, Calprotectin, MMP9/TIMP1 ratio, IL8, BAL neutrophils) were highly correlated and formed the major factor. Pro-inflammatory cytokines in serum, such as IL-6, IL-1β, IFNα, IL-15, MIG, MIP-1α, and TNFα grouped within the second factor, while the more abundant markers in sputum and BAL, such as total protein, HSA and A1AT were combined in factor 3. None of the 3 factors included both serum and sputum or BAL markers, indicating that no significant correlations between these markers exist. This analysis was limited to 29 cases as we had to deal with all missing cases in all compartments. We also performed the same analysis for serum, BAL and ISP separately and included only values from visit 1, which reduced the number of missing cases. The resulting factors were formed by comparable groups of markers within each matrix.

Although a detailed confounder analysis showed that differences in acute smoking and smoking history did not significantly influence our results, we found that mean urine cotinine levels correlated with the same factor as serum MMP9, hematocrit and hemoglobin levels when smoking behavior was included into the above mentioned analysis.

**Figure 2:**
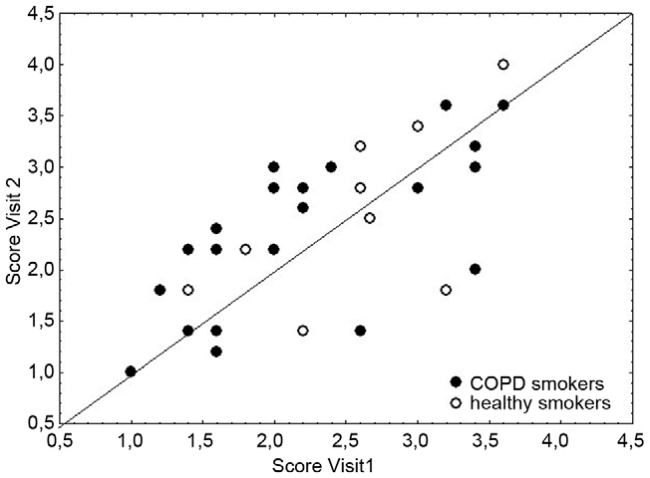
Inflammatory phenotype. Comparison between visits for the scores of the inflammatory phenotype, which were derived from a combination of repeatable sputum fluid phase markers (A1AT, IL6, MMP7, HSA and sputum neutrophils). This combined score shows a better correlation between visits (r = 0.70, p<0.001) as compared to sputum neutrophils alone (see figure 2C).

Finally, we assessed whether it would be possible to define the degree and the different aspects of airway inflammation by multiple lung markers and if such a combined phenotype would be related to a single serum marker. As a large number of different combinations are possible, we focused our analysis on markers related to neutrophilic airway inflammation. Various combinations of BAL markers, including among others calprotectin, IL8, MMP9, and NELA, did not yield a combined score that showed a better repeatability than the respective single markers or a better correlation to a serum marker. Defining an inflammatory phenotype based on the combination of repeatable sputum fluid phase markers (A1AT, IL6, MMP7, HSA and sputum neutrophils) showed a good reproducibility between visits (r = 0.70, p<0.001, Figure 2). Adding additional markers or using only a selection of these markers did not increase the repeatability. However, the mean inflammatory phenotype correlated significantly with mean BAL (r = 0.55, p<0.001) but not with the mean serum calprotectin levels (r = 0.25). The correlation with other serum markers was weak, being best for the mean WBC count (r = 0.5, p = 0.002).

### Alpha-1-antitrypsin

In this study, alpha-1-antitrypsin (A1AT) was analyzed in serum, sputum and in BAL fluid and is the only marker, for which serum concentrations are already clinically used to estimate concentration within the lung and to guide treatment in patients with a known A1AT deficiency. Data comparing A1AT levels between the different compartments are scarce; therefore we used our dataset to test these relationships.

For all subjects, the median (interquartile range, IQR) A1AT concentration was 1.69 (0.53) g/L in serum, 505 (596) µg/L in BAL and 568 (475) µg/L in ISP. A1AT showed a moderate to good reproducibility within each matrix (serum: r = 0.55, p<0.001; BAL: r = 0.72, p<0.001; ISP: r = 0.72, p<0.001, derived from untransformed data, ICC of serum A1AT<0.6, therefore not listed in Table 3). While there was only a weak relationship between mean BAL and ISP A1AT concentrations (r = 0.36, p = 0.03), no relationship was observed between serum and lung concentrations. BAL and ISP A1AT levels did not correlate with neutrophils or serum CRP levels, and only weak correlations between serum A1AT and blood neutrophils (r = 0.31, p = 0.04) or serum CRP levels (r = 0.32, p = 0.04) were observed.

### Safety

The majority of adverse events (AEs) in this study were mild (COPD: 29.2%, healthy smokers: 34.8%) or moderate (COPD: 33.3%, healthy smokers: 17.4%) and were related to a study procedure. Overall 17 of 24 COPD smokers and 12 of 23 healthy smokers reported AEs of which cough was the most frequently used term to describe the symptoms. Two subjects experienced serious adverse events, which were not related to study procedures (1 gastrointestinal bleeding, 1 laryngeal leukoplakia), which led to hospitalization and discontinuing of the study. Overall, the conduct of the study was safe and well tolerated.

## Discussion

We screened a large panel of analytes to find informative and robust biomarkers, which can be used to investigate treatment effects of novel anti-inflammatory compounds for COPD. Our approach was comprehensive with respect to the number of markers, which were studied in all relevant compartments, but very focused by including only age, size, and gender matched smokers with and without moderate COPD. With more than 20 subjects in each group, our study was sufficiently sized and able not only to confirm previous results but also to reveal some currently unknown differences between groups. The collection of samples twice within a period of 4–6 weeks allowed us to assess the repeatability of markers and to create a comprehensive dataset, which was used for further exploration of the interrelationship between markers and systemic and pulmonary compartments. Our analysis revealed that only few and weak correlations between lung and serum markers exist, which does not support the hypothesis that a simple “spill over” of mediators from the lung is responsible for the systemic inflammation observed in COPD. Based on our findings, it is also unlikely that the analysis of serum markers alone will be sufficient to reflect ongoing inflammatory processes within the lung. We describe a lung function independent inflammatory phenotype with an improved repeatability compared to sputum neutrophils alone, which could assist in the understanding of how airway inflammation effects disease progression. Overall, the results of this study, including the comprehensive assay validation data, will help to select markers for clinical and COPD cohort trials.

This study was designed to identify and to test COPD disease specific biomarkers, therefore subjects were carefully matched with respect to smoking history and acute smoking. Although COPD patients reported to smoke slightly more, we did not observe significant differences with respect to urine cotinine. For some analytes, significant differences were found in male subjects only, indicating that gender effects might exist. With only 6 female subjects per group, however, we were not able to reveal gender differences, as. these were recently reported for plasma IL-6, IL-16 and VEGF [20].

Our data confirmed COPD-related increases in the levels of several well-studied serum markers, including CRP, IL-6 [21] [16], and markers associated with neutrophilic airway inflammation [7]. In this respect, it is important to note that, in contrast to ECLIPSE and other studies e.g. [19], we did not compare COPD patients to non-smoking controls, but to healthy smoking subjects.

A novel finding was the upregulation of Calprotectin in BAL fluid. It is considered to be a reliable faecal marker for inflammatory bowel disease. In line with our observation, increased levels of the subunits of calprotectin, S100A8 and S100A9, were found in sputum supernatants of COPD patients [22]. Increased serum levels of vWF in COPD smokers have not been reported so far. It was one of the most significantly increased markers in our data set. This finding, in combination with increased coagulation propensity (decreased PTT), supports the concept that early structural and functional pathophysiological changes of the pulmonary vasculature impair lung perfusion and accompany the development of COPD [3]. In line with this, increased vWF levels have been discussed as a biomarker for endothelial dysfunction in pulmonary arterial hypertension [23]. The increase of vWF in COPD patients was more pronounced in male subjects, which might be due to the fact that vWF levels in healthy women are on average higher than in healthy men [24]. It showed a moderate reproducibility as shown in figure 1 I. Confounder analysis revealed that higher serum vWF levels in COPD patients could not be explained by changed covariates (FEV1 or pack-years, ANCOVA p-values for all confounders <0.01) and no significant association between smoking and vWF levels was found in the ARIC study [24]. Two reports that found vWF to be significantly increased in COPD patients with acute exacerbations [25] [26] support the potential clinical value of this blood marker.

Inflammation is characterized by an increased movement of leucocytes from the microcirculation into the extra-vascular tissue. Cigarette smoke can trigger leukocyte migration and activation [27]. Most likely due to the similar smoking behavior, we neither found evidence for an increased influx of inflammatory cells into the lungs of COPD patients, nor did we observe a higher number of inflammatory cells in their circulation compared to healthy smokers. We found a larger proportion of CD14+ monocytes in the BAL of healthy smokers, especially in the male volunteers, which, to our knowledge, has not been described before. Within both groups, CD14+ monocytes correlated with urine cotinine levels, however, because cotinine levels were similar, the observed difference is unlikely to depend on smoking behavior. Potentially, groups respond differently to oxidative stress or the LPS in cigarette smoke, both of which can cause an increase in the number of CD14+ monocytes in sputum [28].

The recovery of BAL fluid was significantly lower in COPD patients compared to controls (median COPD: 38%, median controls: 73%) which is a well known phenomenon [1]. While overall the correlation between recovery and BAL total cell count was weak, such a relationship was clearly visible in COPD patients and also correlated with the concentration of HSA, potentially indicating that, with a lower recovery, the efficacy of the lavage procedure decreased. Standardizing the mediator concentrations to total protein, however, did not change the observed differences between groups (table 4). In addition, comparable differences between groups were shown for A1AT and HSA in induced sputum samples.

We showed a good reproducibility for a large panel of markers in serum, ISP and BAL when we compared samples collected twice within a period of six weeks, indicating that the marker itself is stable within a subject over this period and that the analysis can be reliably performed. Aaron and coworkers assessed the reproducibility in serum and sputum and even collected 3 samples within the same time period [19]. As reliability criteria the authors assessed intra- and inter-subject variability and computed reference change values for each marker. In line with the data from Aaron et al., we also found serum CRP, serum VEGF and both serum and sputum IL-6 to be reproducible markers. In contrast to Aaron et al., we did not see this for sputum TIMP1, which showed an ICC of just 0.52, or for MPO, most likely due to the fact that we only included smokers and GOLD II patients into our study, resulting in a narrower range of MPO concentrations. Serum IL-6 and CRP were also shown to be repeatable over a one year time period [16] and were shown to increase during COPD exacerbations [12], [29]. In line with our results, TNFα was also found to be stable over a year [16]. The repeatability of sputum neutrophils is well known [30]; its reliability over a one year period was recently shown by Singh et al. [18]. In the ECLIPSE cohort, the repeatability of 15 serum markers was assessed over a 3 month period. However, the results are difficult to compare with our data, as Dickens et al. displayed the percent of values to be within 25% of the respective baseline level [12].

We detected only weak relationships between central (sputum) and peripheral (BAL) airways, which is compatible with other studies [31]. However, more important was the question, whether easily accessible serum markers would be able to reflect the ongoing inflammatory processes within the lung. We first looked at markers that were detected in both compartments. Only calprotectin showed a fairly good relationship between serum and BAL of healthy smokers. It is interesting to note that we did not find evidence for a better correlation between lung and serum in patients with COPD, despite the fact that they did show evidence for systemic inflammation (CRP, IL-6). This does not support the hypothesis that systemic inflammation is caused by a simple “spill-over” of inflammatory markers from the lung into the blood. For A1AT, this lack of relationship between serum and lung could have clinical implications for monitoring treatment. If serum concentrations do not reflect BAL or sputum levels, it appears difficult to estimate lung concentrations from serum data during A1AT supplementation treatment.

Next, we used a factor analysis to structure our data and to test whether any serum marker would be related to a marker detected in the lung. While the markers basically grouped as expected, indicating the validity of our measurements, we did not find serum markers that correlated significantly with any lung marker.

Without any direct relationships between individual serum and lung markers, we choose a third approach and combined different lung markers to develop a score that characterizes the inflammatory phenotype and looked for relationships to systemic markers. This concept links to an observation by Hurst and coworkers who showed that there appears to be a COPD patient phenotype that is more susceptible to exacerbations with stable exacerbation rates that were related to the white blood cell count [29]. This data was very recently confirmed by Agusti et al, showing that those COPD patients with persistent systemic inflammation have increased excerbation rates [32]. The extent of sputum neutrophila as “definition” for a lung inflammatory phenotype in ECLIPSE, did not show a clear relationship to exacerbation rate [18]. We therefore used a combination of robust inflammatory markers in sputum instead of neutrophils to describe the inflammatory phenotype of the subjects in our study. It was shown to be better repeatable than sputum neutrophils alone, possibly because different aspects of inflammation were considered, but it was not related to any serum marker. It would be interesting to learn, whether the approach of combining sputum markers to cover more aspects of inflammation would reveal a relationship to exacerbation rate in larger longitudinal trials like ECLIPSE.

It could be argued, that limiting disease states to GOLD II, which potentially reduced the variability between patients, was responsible for the lack of correlation between lung and serum markers. In the ECLIPSE study no correlation was found between serum IL-6, IL-8, CRP and SP-D and sputum neutrophils [18]. This indicated at least for these serum markers, that investigating COPD patients of all disease stages (GOLD I–IV) does not necessarily reveal correlations between lung and serum inflammatory markers. The baseline serum/plasma measurements showed only weak associations with disease severity in ECLIPSE, with r values being generally below 0.2 [12]. Furthermore, we found quite a wide range of sputum neutrophils in the patients and volunteers of our study, not much different as compared to the GOLD II and GOLD III patients investigated in ECLIPSE.

Novel biomarkers with the ability to predict disease progression will help to test the effectiveness of novel drugs, however, only if the authorities accept that pharmacologically induced changes of these markers are clinically meaningful and expected to predict a difference in symptoms and function in the long term. Our study, with a rather small number of subjects but with well balanced and matched groups of smokers with and without COPD, could contribute to this quest as we assessed a broad panel of potential biomarkers and provided information about their feasibility and repeatability. The tables in the online supplement provide detailed information about sample dilution factors for a large panel of analytes and all relevant matrices. A list of repeatable markers is provided in tables 2 (BAL/sputum) and 3 (serum). The detailed information on the level of markers in different compartments, as well as their variability (for robust markers in table 2/3) can be directly used for sample size and power calculations for future trials. As we did not find any serum marker that sufficiently reflects the inflammatory processes in the airways, we recommend to measure airway inflammation by the least invasive approach, which is currently induced sputum. In addition, our data suggests, that combining different sputum inflammatory markers offers the definition of a robust inflammatory phenotype, which potentially covers more aspects than e.g. looking at the level of neutrophils alone.

Finally, there is a large body of evidence for a role of systemic inflammation in COPD. Serum calprotectin which is related to neutrophilic inflammation as well as serum vWF, an indicator for early structural and functional changes of the pulmonary vasculature could be interesting markers for further exploration.

## Materials and Methods

### Subjects

Twenty-four subjects with moderate COPD (GOLD II) and 23 age- and gender-matched healthy controls were enrolled into this study. All were current smokers (smoking history≥ten pack-years) free of exacerbations or acute infections within four weeks prior to screening and without chronic inflammatory diseases other than COPD. Among other inclusion criteria, a BMI >18 and ≤30 kg/m^2^ and a post-bronchodilator increase in FEV1 ≤15% was required. Subjects or patients with any evidence for a disease that would affect the safety especially during bronchoscopy, with a history of pneumonia within the last 6 month or of asthma were excluded. The study was conducted in accordance with Good Clinical Practice and the Declaration of Helsinki. Subjects gave their written informed consent. The study was approved by the Ethical Committee of Hannover Medical School.

### Study design

**Figure 3:**
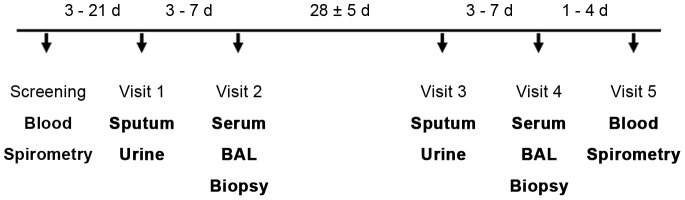
Study design. Blood refers to the sample that was used for hematology and blood chemistry. Serum refers to the sample that was used for biomarker analysis. d = day.

During screening (maximal 3 weeks prior to visit 1), the subject's demographics and medical history was obtained (Figure 3). Blood was drawn for basic hematology and biochemistry. Lung function and ECG were assessed and urine tested for cotinine. Subjects then returned for a total of 5 visits. Visits 1 and 2 (separated by 3–7 d) were followed 28±5 d later by visits 3 and 4 (separated by 3–7 d). During visit 1 and 3, urine was collected and a sputum induction was performed. Bronchoscopy and blood sampling were performed during visits 2 and 4. After a physical examination, blood and urine collection, subjects were discharged from the study in visit 5 (1–4 d after visit 4). All biomarker samples were collected in a fasted state in the morning of the visit day.

### Bronchoscopy and bronchial biopsies

The collection of bronchoalveolar lavage fluid (BAL) and bronchial biopsies was performed as described [33]. Briefly, fiberoptic bronchoscopy was done with standard premedication under topical anesthesia with lidocaine to allow collection of BAL (5×20 mL of sterile saline plus initial 20 mL discard). BAL cells were filtered through a 100 µm filter, centrifuged at 250 g for 10 min, and resuspended in phosphate-buffered saline (PBS). The total count of nucleated cells was performed using a Neubauer hemocytometer. Differential cell counts were performed from cytospin slides, with 300 cells per slide being counted. Total protein was determined according to the method of Bradford [34]. During bronchoscopy, four bronchial biopsies were taken from the segmental level of the right lower lobe using single patient use, radial jaw biopsy forceps (Boston Scientific) that were passed through the working channel of the bronchoscope.

### Induced sputum

Sputum was induced as previously described [35]. Subjects inhaled 3–5% pyrogen-free hypertonic saline from a low output ultrasonic nebulizer. Sputum plugs were selected from the expectorate, stored cooled and processed within 60 min of collection. After homogenisation, sputum supernatants were stored frozen until analysis and cytospin slides were prepared for the differential cell count (at least 400 non-squamous cells).

### Serum sampling

Blood (90 ml) was collected in S-Monovettes® (Sarstedt, Nuembrecht, Germany), allowed to stand for 30 min, and then centrifuged (15 min, 1600 g). Serum was aliquoted and kept frozen at −80°C until analysis.

### Analysis of biomarkers

The analysis was performed by immunoassays (Luminex or ELISA) using commercially available kits (Table S1). Only values above the *limit of detection* (LD = mean of at least 6 blank values plus 3 times standard deviation) were used. Values below the *limit of quantification* (LOQ) were excluded. LOQ was calculated as 80% of lowest detectable standard concentration. All assays were tested for linearity by testing samples in three different dilutions. The acceptance criteria were reached if the calculation of the concentration were in range of 80 to 120% of the expected value. To proof if the assay accurately quantifies the concentration of an analyte which was added to samples, the incremental increase in measured concentration was determined (samples were spiked with high, medium, and low standard). The acceptance criteria were fulfilled if the calculated analyte concentration was in range of 80 to 120% of the value of the added spike concentration. The intra-assay and inter-assay variability were determined and had to be within a range of 80 to 120% of the mean concentration. Measurements had to be successfully performed at least twice. Multiplex bead assays were split for the analysis to enable the use of different optimal dilutions in order to reach the acceptance criteria for the respective analytes (Table S1).

A routine blood chemistry panel (25 parameters) was assessed and 4 parameters were analyzed in urine using mass spectrometry (for details please refer to Text S1). Differential cell counts were performed in BAL and blood on cytospin slides. Cell surface markers (CD3 FITC (Becton Dickinson (BD, Heidelberg, Germany), CD4 PE (BD), CD8 PE (BD), CD14 APC (Beckman Coulter (BC), Krefeld Germany), CD16 PECy7 (BD) and respective isotype controls (BD) were analyzed on BAL cells by flow cytometry. For each analysis 5×10^5^ cells were mixed with an equal volume of goatserum (1∶25 diluted) and incubated for 20 min (4°C). After washing (PBS), pre-determined amounts of antibodies were added and the cells were incubated in the dark for 30 min (4°C). For biotin labelled antibodies an additional incubation step with streptavidin coupled detection antibodies was performed (30 min, 4°C) prior to fixation (Fixation reagent (BC), 1∶40 diluted, 10 min). Next, samples were centrifuged, the supernatant discarded and the cells were resuspended in 600 µL PBS for flow cytometric analysis using a EPICS XL flow cytometer (BC). The data of 10^4^ cells were recorded and analyzed using EXPO 32 and EXPO 32 MultiComp software (BC).

For immunocytochemical detection formalin-fixed biopsies were embedded into paraffin and 3-µm serial sections were cut and mounted on glass slides. The following primary antibodies were used: anti-CD 4 (Novocastra Laboratories Ltd., Newcastle upon Tyne, United Kingdom), anti-CD 8 (Novocastra), anti-CD68 (DakoCytomation, Glostrup, Denmark), and anti-neutrophil elastase (DakoCytomation). Antigen retrieval was performed by protease (Sigma, St. Louis, USA, P-5147) for CD68, CD4 and CD8 in a citrate-buffered solution. Slides were incubated with the primary antibody for 1 h. As secondary antibodies a biotin-SP-conjugated AffiniPure goat-anti-mouse IgG, Fc, subclass1 (Jackson Immunoresearch, USA), or a biotin-SP-conjugated AffiniPure goat-anti-mouse IgG, Fc, subclass 2b (Jackson Immunoresearch) were applied for 30 minutes. Immunostaining was done using alkaline phosphatase streptavidin-biotin (Vector Laboratories Inc, USA) and Fast Red (Fast Red substrate pack, BioGenex, USA). The slides were counterstained with Mayer's hematoxylin (Merck KGaA, Darmstadt, Germany).

Image analysis was performed using a digital camera (ColorView III Soft Imaging System, Olympus, Hamburg, Germany) connected to an automated transmission light microscope (AX70, Olympus) and the image analysis system AnalySIS Five® (Soft Imaging System GmbH, Münster, Germany). Ten images were evaluated of each slide (40-fold magnification). For marker quantification, the analyzed tissue areas were calculated by the software and all cells with positive red labeling were counted interactively on a monitor.

### Data analysis

Prior to the statistical analysis, we corrected the original measurements for plate effects and averaged the duplicated measurements. Based on exploratory data analysis, we standardized the original measurements in BAL and sputum to the total protein content, which accounts for the overall consistency of the sample. The marker concentrations measured in urine were standardized to urine creatinine. In order to identify biomarkers that differed between groups, we conducted an analysis of variance (ANOVA) based on a linear mixed effects model (LME). Differences between groups were reported if the p-values of the parametric as well as of an additional non-parametric analysis were less than 0.05. We conducted a confounder analysis (ANCOVA) for all significant biomarker candidates by extending our original LME models with each confounding factor separately. The following confounders were considered: age, BMI, weight, cigarettes/day, pack-years, urine cotinine, BAL % recovery, FEV1 %pred., FEV1/FVC.

Interrelationships between parameters were investigated by computing the Pearson correlation coefficient. Data displayed in tables 2 and 3 are based on log-transformed data and refer to the following subject numbers: For “ALL” subjects: n = 35–40 (BAL, whole blood, serum), n = 29–33 (sputum), n = 41 (urine); for COPD smokers: n = 16 (FACS data) −20 (BAL, whole blood, serum), n = 20–21 (sputum), n = 20–22 (urine); for healthy smokers: n = 18–20 (BAL, whole blood, serum), n = 10–12 (sputum), n = 20–21 (urine).

Factor analysis was performed using Statistica 9.0 (Statsoft, Tulsa, USA) on both log transformed and standardized datasets. The number of factors (principal components) to be extracted was limited to 3 (Factor rotation: Varimax standard).

To obtain the inflammatory phenotype, we first transformed each selected marker and assigned a score of 1 (lowest values, first quartile of the distribution) to 4 (highest values, fourth quartile). Then we computed the mean value of the resulting scores for different combinations of markers separately for each visit.

The intra-class correlation coefficients (ICC) were derived from one-way ANOVA tables as the ratio of variance among subjects to total variance based on 2 measurements over a 6 week period ([36]: (BMS-WMS/2)/((BMS-WMS/2)+WMS)); BMS = between group mean square, WMS = within group mean square).

## Supporting Information

Figure S1 **Relationship between PTT and serum vWF, separately for smokers with and without COPD. A negative correlation was only observed in healthy smokers.**
(TIF)

Figure S2 **Relationship between CD16+ neutrophils in BAL and the number of neutrophils in bronchial biopsies.** Filled symbols: COPD smoker, open symbols: healthy smoker.
(TIF)

Table S1 **a): ELISA Assays – Vendor and dilution of samples; b) Luminex Assays – Vendor and dilution of samples.**
(DOC)

Table S2 **Cells in BAL fluid.**
(DOC)

Table S3 **BAL fluid mediators.**
(DOC)

Table S4 **Sputum Cells and fluid phase mediators.**
(DOC)

Table S5 **a) Serum mediators analysed by Luminex; b) Serum mediators analysed by other assays.**
(DOC)

Table S6 **Markers in urine.**
(DOC)

Table S7 **Markers bronchial biopsies.**
(DOC)

Table S8 **Markers whole blood.**
(DOC)

Text S1 **Methodology of Urine Analysis.**
(DOC)
