# Supplementary figures and images for: Repeatability of and Relationship between Potential COPD Biomarkers in Bronchoalveolar Lavage, Bronchial Biopsies, Serum, and Induced Sputum
Source: PLoS One. 2012 Oct 4;7(10):e46207. doi: 10.1371/journal.pone.0046207 (PMC3464239; doi:10.1371/journal.pone.0046207)

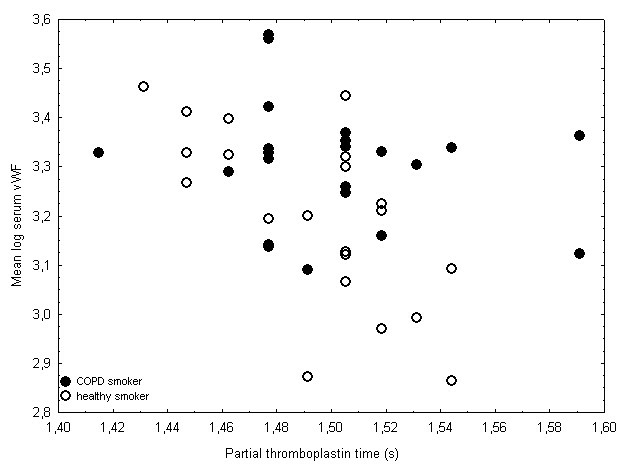

Supplement: Figure S1 — Relationship between PTT and serum vWF, separately for smokers with and without COPD. A negative correlation was only observed in healthy smokers. (TIF) [file pone.0046207.s001.tif]

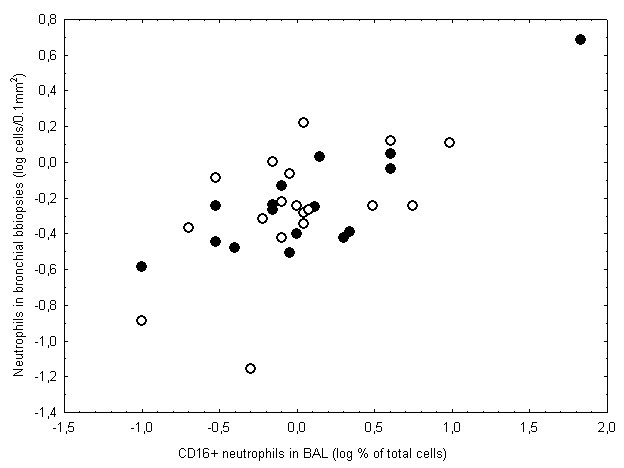

Supplement: Figure S2 — Relationship between CD16+ neutrophils in BAL and the number of neutrophils in bronchial biopsies. Filled symbols: COPD smoker, open symbols: healthy smoker. (TIF) [file pone.0046207.s002.tif]
