# Supplementary material for: Repeatability of and Relationship between Potential COPD Biomarkers in Bronchoalveolar Lavage, Bronchial Biopsies, Serum, and Induced Sputum
Source: PLoS One. 2012 Oct 4;7(10):e46207. doi: 10.1371/journal.pone.0046207 (PMC3464239; doi:10.1371/journal.pone.0046207)
Supplement: Table S1 — a): ELISA Assays – Vendor and dilution of samples; b) Luminex Assays – Vendor and dilution of samples. (DOC) [file pone.0046207.s003.doc]

### Table S1a. ELISA Assays – Vendor and dilution of samples

| **Analyte** | **Vendor** | **Ordernr.** | **Sample Dilution** |
| --- | --- | --- | --- |
| **Panel Serum** |  |  |  |
| Leukotriene (LTB4) | AssayDesigns | 900-068 | **1:40** |
| vWF | Assay Pro | EV2030-1 | **1:60** |
| Calprotectin | HyCult | HK325 | **1:100** |
| EGF-R | Calbiochem | CBA018 | **1:40** |
| Serotonin | IBL Hamburg | RE59121 | **1:2** |
| NELA | HyCult | HK319 | **1:200** |
| TIMP-1 | R&D | DTM100 | **1:40** |
| TIMP-2 | R&D | DTM200 | **1:40** |
| a1-Antitrypsin | Immundiagnostik | K6750 | **1:80.000** |
| TGF-beta (bound) | Promega | G7591 | **1:20** |
| TGF-beta (free) | Promega | G7591 | **1:4** |
| VEGF | R&D | DVE00 | **1:2** |
| IL-6 | R&D | SS600B | **1:3** |
| **Panel BALF** |  |  |  |
| EGF-R | Calbiochem | CBA018 | **1:2** |
| Serotonin | IBL Hamburg | RE59121 | **1:2** |
| Calprotectin | HyCult | HK325 | **1:8** |
| NELA | HyCult | HK319 | **1:20** |
| a1-Antitrypsin | Immundiagnostik | K6750 | **1:20** |
| TIMP-1 | R&D | DTM100 | **1:2** |
| TotalProtein | Pierce | 1856210 | **1:5+1:10** |
| HSA | Bethyl | E80-129 | **1:200** |
| **Panel Sputum** |  |  |  |
| Serotonin | IBL Hamburg | RE59121 | **1:4** |
| Leukotriene (LTB4) | AssayDesigns | 900-068 | **1:40** |
| TIMP-1 | R&D | DTM100 | **1:40** |
| TIMP-2 | R&D | DTM200 | **1:6** |
| TGF-beta (bound) | Promega | G7591 | **1:8** |
| TGF-beta (free) | Promega | G7591 | **1:40** |
| NELA | HyCult | HK319 | **1:2.000** |
| a1-Antitrypsin | Immundiagnostik | K6750 | **1:200** |
| TotalProtein | Pierce | 1856210 | **1:25+1:50** |
| HSA | Bethyl | E80-129 | **1:1.000** |

### Table S1b. Luminex Assays – Vendor and dilution of samples

| **Kit** | **Vendor** | **Ordernr.** | **Analyte** | **Sample Dilution** |
| --- | --- | --- | --- | --- |
| **Panel Serum** |  |  |  |  |
| 25-Plex | Biosource | LHC0009 | IFNalpha, IFNgamma, IL-1beta, IL-2, IL-2R, IL-5, IL-6, IL-7, IL-8, IL-12p40/p70, IL-13, IL-15, MIG, MIP-1alpha, MIP-1beta, TNFalpha | **undiluted** |
| Custom Duplex | Biosource | LHG0061/LHG0021 | EGF / FGF basic | **undiluted** |
| Single-Plex | R&D | LUB672 | I-TAC | **1:2** |
| Custom 3-Plex | Biosource | LHC2231/LHC2011/ LHC0171 | Eotaxin / GM-CSF / IL-17 | **1:3** |
| Single-Plex | Biosource | LHC0101 | IL-10 | **1:3** |
| Death Receptor 3-Plex | Biosource | LHC0006 | TNF-R1, TNF-R2, DR5 | **1:3** |
| Single-Plex | Biosource | LHG 0071 | HGF | **1:3** |
| Single-Plex | Biosource | LHG0111 | VEGF | **1:5** |
| Single-Plex | Biosource | LHC0041 | IL-4 | **1:5** |
| Custom Duplex | Biosource | LHC1011/LHC0711 | MCP-1 / IL-1RA | **1:10** |
| Single-Plex | Linco | HCYTO-60K-01 | IP-10 | **1:10** |
| Single-Plex | Upstate | 46-142 | PDGF AA | **1:10** |
| Single-Plex | R&D | LMP902 | MMP-2 | **1:20** |
| Single-Plex | Linco | HCYTO-60K-01 | E-Selectin | **1:25** |
| Single-Plex | Upstate | 46-143 | PDGF AB/BB | **1:50** |
| Single-Plex | Biosource | LHC1031 | RANTES | **1:50** |
| Single-Plex | R&D | LMP901 | MMP-1 | **1:50** |
| Duplex | Linco | HCVD1-67AK-02 | ICAM, MPO | **1:100** |
| Single-Plex | Linco | HCVD1-67AK-01 | VCAM | **1:250** |
| Single-Plex | R&D | LMP911 | MMP-9 | **1:500** |
| Single-Plex | Biosource | LHP0031 | CRP | **1:500** |
| **Panel BALF** |  |  |  |  |
| 25-Plex | Biosource | LHC0009 | IFNalpha, IFNgamma, IL-4, IL-5, IL-6, IL-7, IL-8, IL-10, IP-10, MIP-1beta, TNFalpha, GM-CSF | **undiluted** |
| Custom 3-Plex | R&D | LMP901/LMP911 | MMP-1 / MMP-9 / MMP-12 | **1:5** |
| Single-Plex | R&D | LMP902 | MMP-2 | **1:2** |
| 25-Plex | Biosource | LHC0009 | Eotaxin, IL-1beta,IL-1RA, IL-2, IL-2R, IL-12p40/p70, IL-13, IL-15, IL-17, MIG, MIP-1alpha, RANTES | **1:3** |
| Death Receptor 3-Plex Growth Factor 4-Plex | Biosource | LHC0006/LHC0004 | TNF-R1, TNF-R2, DR5 EGF, FGFbasic, G-CSF, VEGF | **1:3** |
| Single-Plex | R&D | LUB672 | I-TAC | **1:5** |
| Single-Plex | Linco | HCVD1-67AK-01 | MPO | **1:40** |
| Single-Plex | Biosource | LHP0031 | CRP | **1:5** |
| **Panel Sputum** |  |  |  |  |
| Custom Duplex | Biosource | LHC0171/LHC3011 | IL-17 / TNFalpha | **undiluted** |
| 25-Plex | Biosource | LHC0009 | IL-1beta, IL-2, IL-2R, IL-6, IL-15, IP-10, MCP-1, MIP-1alpha | **1:2** |
| Single-Plex | Biosource | LHC1031 | RANTES | **1:5** |
| Single-Plex | Biosource | LHC0081 | IL-8 | **1:50** |
| Single-Plex | Biosource | LHC0711 | IL-1RA | **1:100** |
| Single-Plex | Biosource | LHP0031 | CRP | **undiluted** |
| Single-Plex | R&D | LMP919 | MMP-12 | **undiluted** |
| Single-Plex | R&D | LMP901 | MMP-1 | **1:2** |
| Custom Duplex | R&D | LMP513/LMP511 | MMP-3 / MMP-13 | **1:5** |
| Single-Plex | R&D | LMP902 | MMP-2 | **1:10** |
| Single-Plex | R&D | LMP907 | MMP-7 | **1:20** |
| Custom Duplex | R&D | LMP908/LMP911 | MMP-8 / MMP-9 | **1:200** |
| Single-Plex | Biosource | LHG0061 | EGF | **1:3** |
