# Supplementary material for: Repeatability of and Relationship between Potential COPD Biomarkers in Bronchoalveolar Lavage, Bronchial Biopsies, Serum, and Induced Sputum
Source: PLoS One. 2012 Oct 4;7(10):e46207. doi: 10.1371/journal.pone.0046207 (PMC3464239; doi:10.1371/journal.pone.0046207)
Supplement: Table S2 — Cells in BAL fluid. (DOC) [file pone.0046207.s004.doc]

Table S2: Cells in BAL fluid

| **Analyte** | **M** | **Unit** | **First visit** | | **Second visit** | | **LME-ANOVA** |
| --- | --- | --- | --- | --- | --- | --- | --- |
|  |  |  | **healthy smokers** | **COPD smokers** | **healthy smokers** | **COPD smokers** | **p-value** |
| CD3+ LY | F | %TC | 0.3 (0.2-0.5) | 0.1 (0.1-0.2) | 0.2 (0.2-0.3) | 0.2 (0.1-0.2) | 0,041 |
| CD3+ LY | F | 10^3^/mL | 1.0 (0.3-1.6) | 0.4 (0.2-0.6) | 0.4 (0.3-1.0) | 0.4 (0.2-1.2) | 0,337 |
| CD4+ LY | F | %TC | 0.2 (0.2-0.3) | 0.1 (0.1-0.1) | 0.2 (0.1-0.3) | 0.1 (0.1-0.1) | 0,051 |
| CD4+ LY | F | 10^3^/mL | 0.5 (0.2-1.1) | 0.3 (0.1-0.5) | 0.3 (0.2-0.8) | 0.2 (0.1-0.2) | 0,053 |
| CD8+ LY | F | %TC | 0.2 (0.1-0.4) | 0.1 (0.1-0.1) | 0.1 (0.1-0.2) | 0.2 (0.1-0.2) | 0,260 |
| CD8+ LY | F | 10^3^/mL | 0.5 (0.1-0.7) | 0.3 (0.1-0.4) | 0.2 (0.1-0.4) | 0.3 (0.1-0.5) | 0,239 |
| CD16+ NG | F | %TC | 0.9 (0.5-2.2) | 1.1 (0.6-3.0) | 0.9 (0.6-1.2) | 0.9 (0.4-2.0) | 0,349 |
| CD16+ NG | F | 10^3^/mL | 1.7 (1.0-3.5) | 2.8 (1.1-5.8) | 2.2 (1.2-4.0) | 1.7 (0.8-3.4) | 0,736 |
| MACROPHAGES | Diff. | % | 95.3 (92.6-97.2) | 96.0 (92.9-97.2) | 95.2 (92.5-95.8) | 94.4 (91.5-96.3) | 0,331 |
| NEUTROPHILS | Diff. | % | 0.8 (0.5-1.7) | 0.7 (0.5-1.5) | 0.9 (0.3-2.8) | 1.3 (0.6-1.8) | 0,645 |
| EOSINOPHILS | Diff. | % | 0.5 (0.3-1.2) | 0.6 (0.3-1.9) | 0.8 (0.5-1.0) | 0.6 (0.4-2.4) | 0,386 |
| EPITHELIAL CELLS | Diff. | % | 0.5 (0.4-0.5) | 0.3 (0.3-2.7) | 0.3 (0.3-0.5) | 1.0 (0.3-2.0) | 0,142 |
| LYMPHOCYTES | Diff. | % | 0.8 (0.5-2.1) | 0.7 (0.5-1.3) | 1.0 (0.5-2.1) | 0.7 (0.3-1.1) | 0,180 |
| MONOCYTES | Diff. | % | 2.0 (1.3-3.0) | 1.8 (1.3-2.8) | 2.9 (2.0-3.6) | 2.0 (1.4-2.7) | 0,122 |
| Data presented as median (IQR), LME-ANOVA p-value: COPD smokers vs. healthy smokers. M=Method of analysis, Diff.=differential cell count, TC= total cellcount, F= FACS, Ly=Lymphocytes, NG=Neutrophils | | | | | | | |
