# Supplementary material for: Repeatability of and Relationship between Potential COPD Biomarkers in Bronchoalveolar Lavage, Bronchial Biopsies, Serum, and Induced Sputum
Source: PLoS One. 2012 Oct 4;7(10):e46207. doi: 10.1371/journal.pone.0046207 (PMC3464239; doi:10.1371/journal.pone.0046207)
Supplement: Table S3 — BAL fluid mediators. (DOC) [file pone.0046207.s005.doc]

Table S3: BAL fluid mediators

| **Analyte** | **M** | **Unit** | **First visit** | | **Second visit** | | **LME-ANOVA** |
| --- | --- | --- | --- | --- | --- | --- | --- |
|  |  |  | **healthy smokers** | **COPD smokers** | **healthy smokers** | **COPD smokers** | **p-value** |
| Calprotectin | E | ng/ml | 59.4 (33.9-84.5) | 64.0 (50.3-77.3) | 58.9 (36.7-77.6) | 58.3 (43.2-78.1) | 0,259 |
| IGFBP-1 | Mm | ng/ml | 0.1 (0.1-0.2) | 0.1 (0.1-0.2) | 0.1 (0.1-0.2) | 0.1 (0.1-0.2) | m: 0,11, f:0,279 |
| IGFBP-2 | Me | ng/ml | 3.1 (2.5-4.6) | 3.0 (1.9-4.0) | 4.0 (2.4-4.8) | 3.3 (2.4-4.9) | 0,499 |
| IL-13 | L | pg/ml | 57.2 (46.0-66.4) | 50.3 (46.5-66.7) | 101.5 (61.2-146.8) | 77.6 (61.7-121.4) | 0,35 |
| IL-1Ra | L | pg/ml | 66.6 (20.4-108.8) | 64.4 (46.7-80.6) | 134.4 (87.7-198.5) | 121.2 (86.4-190.3) | 0,688 |
| IL-2 | L | pg/ml | 2.4 (1.2-5.4) | 1.1 (0.2-4.0) | 20.9 (1.9-30.2) | 7.4 (2.0-24.4) | 0,701 |
| IL-6 | E | pg/ml | 11.5 (10.1-12.3) | 9.6 (7.4-11.6) | 7.6 (6.3-8.9) | 11.4 (9.2-11.9) | 0,966 |
| IL-8 | L | pg/ml | 18.3 (12.4-39.5) | 23.5 (15.4-42.3) | 19.4 (13.7-34.8) | 26.8 (16.7-51.1) | 0,117 |
| IL-8 | E | pg/ml | 57.7 (40.1-92.2) | 83.1 (59.9-103.5) | 48.4 (38.3-65.7) | 62.5 (41.7-83.2) | 0,233 |
| IP-10 | L | pg/ml | 2.8 (2.2-3.3) | 2.8 (2.2-3.3) | 1.3 (0.8-3.2) | 1.7 (0.8-2.7) | 0,476 |
| LBP | E | µg/ml | 0.6 (0.5-0.7) | 0.7 (0.6-0.7) | 0.7 (0.6-0.8) | 0.6 (0.5-0.7) | 0,926 |
| MCP-1 | E | pg/ml | 46.7 (42.0-71.6) | 49.4 (41.8-71.4) | 53.5 (38.6-61.3) | 59.8 (49.2-137.7) | 0,306 |
| MIP-1alpha | L | pg/ml | 103.8 (86.6-118.3) | 98.5 (80.7-103.8) | 1219.7 (77.6-1219.7) | 145.3 (55.4-1219.7) | 0,142 |
| MMP-9 | L | pg/ml | 1976 (752-3755) | 1552 (1193-2912) | 2062 (681-4439) | 2238 (651-4033) | 0,740 |
| MPO | L | pg/ml | 3428 (2919-7036) | 3479 (2558-5235) | 5235 (5215-9866) | 4298 (1624-5562) | 0,052 |
| NELA | E | pg/ml | 14107 (10773-26689) | 10938 (9202-31542) | 9202 (8046-22517) | 9202 (8046-16868) | 0,610 |
| RANTES | L | pg/ml | 61.2 (40.5-86.1) | 59.8 (33.9-72.5) | 97.7 (54.2-149.0) | 79.5 (56.5-111.2) | 0,349 |
| total-protein | E | µg/ml | 69.4 (53.3-92.3) | 52.7 (45.1-87.6) | 81.1 (54.0-96.9) | 68.6 (48.8-95.8) | m: 0,021, f:0,09 |
| IGFBP-1/TP | Me | ng/µg | 0.0 (0.0-0.0) | 0.0 (0.0-0.0) | 0.0 (0.0-0.0) | 0.0 (0.0-0.0) | 0,450 |
| IGFBP-2/TP | Me | ng/µg | 0.0 (0.0-0.1) | 0.1 (0.0-0.1) | 0.0 (0.0-0.1) | 0.1 (0.0-0.1) | m: 0,13, f:0,096 |
| IL-13/TP | L | pg/µg | 0.8 (0.5-1.2) | 0.9 (0.7-1.3) | 1.3 (0.7-2.5) | 1.4 (0.8-2.2) | m: 0,099, f:0,10 |
| IL-1Ra/TP | L | pg/µg | 0.9 (0.3-1.4) | 1.2 (0.7-1.6) | 1.8 (1.0-2.9) | 2.0 (1.0-3.0) | m: 0,06, f:0,22 |
| IL-2/TP | L | pg/µg | 0.0 (0.0-0.1) | 0.0 (0.0-0.1) | 0.3 (0.0-0.4) | 0.1 (0.0-0.3) | 0,787 |
| IP-10/TP | L | pg/µg | 0.0 (0.0-0.1) | 0.0 (0.0-0.1) | 0.0 (0.0-0.1) | 0.0 (0.0-0.0) | 0,946 |
| LBP/TP | E | µg/µg | 0.0 (0.0-0.0) | 0.0 (0.0-0.0) | 0.0 (0.0-0.0) | 0.0 (0.0-0.0) | m: 0,048, f:0,31 |
| MIP-1alpha/TP | L | pg/µg | 1.5 (0.9-2.1) | 1.7 (1.1-2.1) | 33.6 (1.0-33.6) | 3.0 (0.9-33.6) | 0,408 |
| MMP-9/TP | L | pg/µg | 22.7 (8.7-56.1) | 30.0 (19.3-49.0) | 27.3 (6.7-58.5) | 26.1 (13.1-64.6) | m: 0,10, f:0,39 |
| MPO/TP | L | pg/µg | 44.9 (31.0-89.0) | 50.7 (31.0-90.6) | 61.1 (50.7-103.0) | 49.8 (25.7-54.0) | 0,214 |
| NELA/TP | E | pg/µg | 149.4 (109.6-350.9) | 128.1 (66.3-379.2) | 109.8 (91.2-299.1) | 152.4 (109.6-261.1) | m: 0,21, f:0,127 |
| RANTES/TP | L | pg/µg | 0.8 (0.5-1.2) | 1.0 (0.6-1.3) | 1.3 (0.7-1.7) | 1.4 (0.6-1.6) | m: 0,10, f:0,02 |
| Data presented as median (IQR). LME-ANOVA p-value: COPD smokers vs. healthy smokers. M=Method, TP=normalized to total protein, E=ELISA, L=Luminex Me=Mediagnost | | | | | | | |
