# Supplementary material for: Repeatability of and Relationship between Potential COPD Biomarkers in Bronchoalveolar Lavage, Bronchial Biopsies, Serum, and Induced Sputum
Source: PLoS One. 2012 Oct 4;7(10):e46207. doi: 10.1371/journal.pone.0046207 (PMC3464239; doi:10.1371/journal.pone.0046207)
Supplement: Table S4 — Sputum Cells and fluid phase mediators. (DOC) [file pone.0046207.s006.doc]

Table S4: Sputum Cells and fluid phase mediators

| **Analyte** | **M** | **Unit** | **First visit** | | **Second visit** | | **LME-ANOVA** |
| --- | --- | --- | --- | --- | --- | --- | --- |
|  |  |  | **healthy smokers** | **COPD smokers** | **healthy smokers** | **COPD smokers** | **p-value** |
| TOTAL CELL COUNT | Diff | 10^4^/g | 120.5 (76.2-245.3) | 77.3 (52.4-165.0) | 118.2 (73.5-152.4) | 78.1 (33.7-114.0) | 0,279 |
| MACROPHAGES | Diff | % | 7.3 (5.2-14.5) | 10.0 (8.6-11.9) | 10.6 (4.7-15.3) | 8.5 (4.7-12.2) | 0,951 |
| NEUTROPHILS | Diff | % | 56.2 (32.0-71.7) | 53.8 (41.2-71.3) | 40.9 (23.9-60.3) | 53.5 (41.5-64.7) | m: 0,20, f:0,078 |
| EOSINOPHILS | Diff | % | 0.5 (0.3-1.0) | 1.0 (0.7-2.1) | 1.0 (0.8-1.8) | 1.0 (0.5-2.3) | 0,215 |
| LYMPHOCYTES | Diff | % | 1.3 (0.7-2.4) | 2.4 (1.9-4.7) | 2.4 (1.1-3.9) | 2.8 (1.2-4.6) | 0,026 |
| MONOCYTES | Diff | % | 2.1 (1.0-3.5) | 2.2 (1.1-3.6) | 1.3 (0.8-2.4) | 2.0 (1.2-3.0) | m: 0,138, f:0,018 |
| EPITHELIAL CELLS | Diff | % | 28.8 (8.5-56.6) | 30.2 (7.5-40.8) | 37.3 (20.4-46.5) | 28.8 (9.5-41.0) | m: 0,01, f:0,011 |
| total-protein | E | µg/ml | 326.1 (279.4-515.4) | 329.7 (273.6-362.2) | 324.8 (280.2-578.2) | 330.4 (308.7-360.6) | 0,504 |
| IGFBP-1 | Me | ng/ml | 0.8 (0.5-0.8) | 0.7 (0.5-0.9) | 0.4 (0.4-0.6) | 0.4 (0.3-0.7) | m: 0,30, f:0,26 |
| IL-6 | L | pg/ml | 141.1 (37.1-202.8) | 50.3 (35.9-92.3) | 58.9 (32.6-200.3) | 54.1 (32.0-95.1) | 0,150 |
| IP-10 | L | pg/ml | 63.4 (32.9-118.7) | 52.2 (37.5-126.2) | 33.2 (17.4-93.4) | 52.1 (24.6-74.9) | 0,895 |
| MCP-1 | L | pg/ml | 450.2 (349.7-596.3) | 450.2 (288.7-637.9) | 339.1 (203.4-571.0) | 370.2 (273.4-527.0) | 0,874 |
| MMP 7 | L | pg/ml | 2389 (1248-7910) | 3450 (1323-6076) | 3309 (1401-14480) | 3375 (1955-8774) | 0,964 |
| MMP-1 | L | pg/ml | 25.7 (9.5-39.4) | 29.9 (18.3-44.4) | 12.6 (6.0-24.3) | 22.2 (8.3-39.7) | 0,606 |
| MMP-12 | L | pg/ml | 106.5 (91.3-156.3) | 93.8 (70.5-127.1) | 57.2 (40.4-115.2) | 93.5 (66.6-130.1) | 0,998 |
| MMP-9 | L | pg/ml | 65960 (3126-164444) | 95925 (34201-160457) | 167894 (55504-269638) | 106783 (69292-164089) | m: 0,14, f:0,26 |
| TIMP-1 | E | ng/ml | 76.2 (50.0-112.8) | 92.6 (61.6-127.6) | 99.5 (35.7-152.6) | 97.5 (71.4-125.5) | 0,689 |
| TIMP-2 | E | ng/ml | 6.5 (3.2-9.4) | 5.4 (3.6-8.3) | 5.3 (2.0-6.8) | 4.3 (2.9-4.8) | 0,883 |
| IGFBP-1/TP | Me | ng/µg | 0.0 (0.0-0.0) | 0.0 (0.0-0.0) | 0.0 (0.0-0.0) | 0.0 (0.0-0.0) | 0,757 |
| IL-6/TP | L | pg/µg | 0.3 (0.1-0.4) | 0.2 (0.1-0.3) | 0.2 (0.1-0.4) | 0.2 (0.1-0.3) | 0,311 |
| IP-10/TP | L | pg/µg | 0.2 (0.1-0.2) | 0.2 (0.1-0.3) | 0.1 (0.1-0.2) | 0.2 (0.1-0.2) | 0,405 |
| MCP-1/TP | L | pg/µg | 0.9 (0.8-1.2) | 1.1 (0.8-1.7) | 1.1 (0.8-1.3) | 1.1 (0.8-1.5) | 0,417 |
| MMP 3/TP | L | pg/µg | 0.1 (0.1-0.1) | 0.1 (0.1-0.1) | 0.1 (0.1-0.2) | 0.1 (0.0-0.1) | m: 0,023, f:0,43 |
| MMP 7/TP | L | pg/µg | 10.0 (3.6-17.7) | 10.1 (5.1-22.6) | 11.8 (5.1-27.8) | 13.0 (6.5-28.6) | 0,627 |
| MMP-1/TP | L | pg/µg | 0.1 (0.0-0.1) | 0.1 (0.1-0.1) | 0.0 (0.0-0.0) | 0.1 (0.0-0.1) | 0,299 |
| MMP-12/TP | L | pg/µg | 0.3 (0.2-0.6) | 0.3 (0.2-0.4) | 0.2 (0.1-0.2) | 0.3 (0.2-0.4) | 0,582 |
| MMP-9/TP | L | pg/µg | 202.7 (11.1-390.2) | 316.8 (195.5-433.4) | 430.0 (265.6-771.5) | 368.0 (247.1-487.2) | m: 0,066, f:0,10 |
| TIMP-1/TP | E | ng/µg | 0.2 (0.2-0.3) | 0.3 (0.2-0.4) | 0.2 (0.1-0.4) | 0.3 (0.2-0.4) | 0,114 |
| TIMP-2/TP | E | ng/µg | 0.0 (0.0-0.0) | 0.0 (0.0-0.0) | 0.0 (0.0-0.0) | 0.0 (0.0-0.0) | 0,426 |
| Data presented as median (IQR). LME-ANOVA p-value: COPD smokers vs. healthy smokers. M=Method of analysis, TP=normalized to total protein, E=ELISA, L=Luminex, Me=Mediagnost | | | | | | | |
