# Supplementary material for: Repeatability of and Relationship between Potential COPD Biomarkers in Bronchoalveolar Lavage, Bronchial Biopsies, Serum, and Induced Sputum
Source: PLoS One. 2012 Oct 4;7(10):e46207. doi: 10.1371/journal.pone.0046207 (PMC3464239; doi:10.1371/journal.pone.0046207)
Supplement: Table S5 — a) Serum mediators analysed by Luminex; b) Serum mediators analysed by other assays. (DOC) [file pone.0046207.s007.doc]

Table S5: a) Serum mediators analysed by Luminex

| **Analyte** | **M** | **Unit** | **First visit** | | **Second visit** | | **LME-ANOVA** |
| --- | --- | --- | --- | --- | --- | --- | --- |
|  |  |  | **healthy smokers** | **COPD smokers** | **healthy smokers** | **COPD smokers** | **p-value** |
| EGF | L | pg/ml | 141.6 (122.5-171.8) | 139.5 (93.0-216.0) | 134.5 (103.0-176.8) | 123.8 (85.5-171.7) | 0,847 |
| Eotaxin | L | pg/ml | 227.6 (199.3-252.6) | 263.3 (184.0-332.9) | 220.5 (179.3-292.9) | 244.6 (190.3-336.7) | 0,159 |
| E-Selectin | L | pg/ml | 6841.5 (4739.2-12866.7) | 7775.9 (5055.9-13465.2) | 13898.0 (7957.0-21914.6) | 15391.6 (10312.2-25186.9) | m: 0,13, f:0,12 |
| ICAM-1 | L | pg/ml | 111545 (79032-130324) | 112829 (90344-152231) | 121361 (77225-164424) | 138030 (104535-181154) | 0,101 |
| IFN-alpha | L | pg/ml | 48.1 (39.7-59.1) | 60.8 (38.8-86.7) | 47.9 (40.5-60.6) | 63.7 (47.0-88.2) | 0,037 |
| IFN-gamma | L | pg/ml | 24.4 (17.1-29.5) | 22.8 (18.7-42.9) | 24.5 (16.9-29.9) | 32.1 (19.2-49.4) | 0,103 |
| IL-12p40/p70 | L | pg/ml | 179.6 (170.2-214.3) | 203.0 (163.7-227.7) | 166.3 (150.7-193.2) | 168.1 (147.8-211.7) | m: 0,14, f:0,109 |
| IL-13 | L | pg/ml | 10.0 (7.3-15.3) | 19.2 (10.0-26.7) | 18.9 (12.1-29.4) | 26.3 (17.1-33.6) | 0,109 |
| IL-15 | L | pg/ml | 31.7 (27.2-47.2) | 36.9 (29.8-56.0) | 33.1 (22.6-41.4) | 43.3 (31.9-65.9) | 0,157 |
| IL-1beta | L | pg/ml | 294.0 (94.8-565.5) | 518.7 (204.5-927.8) | 314.0 (124.2-568.8) | 582.7 (319.0-898.6) | 0,211 |
| IL-2 | L | pg/ml | 3.9 (2.9-10.5) | 6.5 (3.9-14.6) | 3.4 (2.9-14.1) | 10.2 (4.7-21.9) | 0,298 |
| IL-2R | L | pg/ml | 524.2 (457.1-659.0) | 576.7 (510.4-762.8) | 458.9 (402.7-545.9) | 526.5 (407.6-685.3) | 0,213 |
| IL-7 | L | pg/ml | 34.5 (30.6-44.4) | 46.2 (33.7-57.1) | 32.8 (23.6-40.7) | 45.4 (35.3-60.9) | 0,064 |
| IL-8 | L | pg/ml | 33.3 (22.3-39.0) | 28.5 (16.5-36.8) | 26.0 (17.0-31.3) | 21.4 (15.9-28.2) | 0,554 |
| IP-10 | L | pg/ml | 60.1 (39.2-99.0) | 74.9 (46.0-124.5) | 56.4 (32.5-79.5) | 55.3 (37.5-86.1) | 0,391 |
| I-TAC | L | pg/ml | 72.1 (51.1-87.4) | 79.5 (59.0-89.6) | 63.9 (38.5-74.3) | 52.5 (41.7-80.4) | 0,617 |
| MCP-1 | L | pg/ml | 2879 (1913-3521) | 3789 (2360-5656) | 2879 (2043-3784) | 4092 (2989-5000) | 0,056 |
| MIG | L | pg/ml | 94.0 (64.9-109.0) | 95.3 (73.8-123.2) | 94.8 (74.2-122.8) | 107.7 (70.9-175.7) | 0,166 |
| MIP-1alpha | L | pg/ml | 64.4 (55.9-77.8) | 70.5 (61.6-85.8) | 60.0 (53.8-69.2) | 74.4 (60.6-102.0) | 0,092 |
| MIP-1beta | L | pg/ml | 143.9 (126.4-192.8) | 162.0 (120.2-242.8) | 115.5 (98.7-174.9) | 139.3 (112.1-195.7) | m: 0,05, f:0,23 |
| MMP-1 | L | pg/ml | 4876 (2409-6335) | 5054 (2648-8599) | 5381 (3510-7487) | 5581 (2955-9018) | 0,749 |
| MMP-2 | L | pg/ml | 128893 (95327-159044) | 138947 (120036-272722) | 182134 (135635-380720) | 286291 (166406-392088) | 0,052 |
| MMP-9 | L | ng/ml | 450.9 (371.0-562.5) | 619.6 (503.7-713.4) | 588.7 (416.4-731.9) | 567.9 (439.1-821.4) | 0,050 |
| MPO | L | pg/ml | 35395 (23153-54376) | 33786 (20727-71906) | 38237 (25105-62761) | 73935 (44877-106613) | 0,088 |
| PDGF-AA | L | pg/ml | 11953 (8320-15122) | 11504 (9470-14192) | 9783 (7603-13772) | 10045 (7822-11539) | 0,623 |
| PDGF-AB/BB | L | pg/ml | 16394 (11883-19571) | 15999 (11745-20620) | 12455 (9806-16463) | 12162 (9436-16572) | 0,885 |
| RANTES | L | pg/ml | 36981 (29331-62553) | 45200 (29148-63531) | 49276 (38321-63010) | 52059 (33696-66105) | m: 0,15, f:0,14 |
| TNF-alpha | L | pg/ml | 4.6 (3.7-5.9) | 5.4 (4.1-8.2) | 4.1 (3.2-5.7) | 7.0 (4.0-9.1) | 0,046 |
| TNF-R1 | L | pg/ml | 671 (562-798) | 876 (594-1090) | 989 (675-1427) | 1154 (757-1422) | 0,192 |
| TNF-R2 | L | pg/ml | 1356 (1090-1735) | 1473 (1243-2137) | 2343 (1177-2994) | 1501 (1053-3027) | 0,831 |
| VCAM-1 | L | pg/ml | 4289607 (285843-6266669) | 418235 (226848-7908586) | 390207 (221756-1398034) | 424450 (266326-844302) | 0,362 |
| Data presented as median (IQR). LME-ANOVA p-value: COPD smokers vs. healthy smokers. M=Method of analysis, TP=total protein, L=Luminex | | | | | | | |

Table S5: b) Serum mediators analysed by other assays

| **Analyte** | **M** | **Unit** | **First visit** | | **Second visit** | | **LME-ANOVA** |
| --- | --- | --- | --- | --- | --- | --- | --- |
|  |  |  | **healthy smokers** | **COPD smokers** | **healthy smokers** | **COPD smokers** | **p-value** |
| CREATININE | EB | mg/dl | 0.8 (0.7-0.9) | 0.8 (0.7-0.9) | 0.8 (0.8-0.9) | 0.8 (0.7-0.9) | m: 0,72, f:0,061 |
| HSA | EB | g/dl | 4.2 (4.1-4.3) | 4.3 (4.0-4.4) | 4.3 (4.1-4.4) | 4.1 (4.0-4.4) | 0,543 |
| LBP | E | µg/ml | 5.9 (5.1-6.7) | 6.4 (5.7-7.6) | 6.4 (5.9-7.1) | 7.5 (6.6-8.5) | 0,136 |
| Calprotectin | E | ng/ml | 403.7 (194.3-491.2) | 453.5 (382.0-523.0) | 380.9 (192.5-465.1) | 470.4 (332.2-522.0) | 0,209 |
| EGF-R | E | pg/ml | 45739.5 (41321.0-50139.8) | 45258.5 (41596.3-49333.8) | 46750.0 (41991.3-51148.0) | 43187.5 (40393.0-49344.5) | 0,480 |
| NELA | E | ng/ml | 532.1 (362.0-729.8) | 729.2 (402.8-118.3) | 646.5 (394.1-944.2) | 719.3 (508.2-118.7) | 0,093 |
| Serotonin | E | ng/ml | 196.3 (159.8-232.8) | 189.8 (153.3-216.1) | 212.4 (176.2-231.9) | 218.1 (177.1-253.2) | 0,337 |
| TGF-beta_bound | E | pg/ml | 15932.5 (12926.0-29723.8) | 15306.0 (14001.3-27063.5) | 18265.0 (14866.0-24320.3) | 18806.0 (16720.5-23827.8) | 0,866 |
| TIMP-1 | E | ng/ml | 152.0 (130.6-174.7) | 166.8 (145.7-186.7) | 155.6 (138.2-180.0) | 166.5 (139.0-190.7) | 0,363 |
| TIMP-2 | E | ng/ml | 79.9 (64.6-85.7) | 81.5 (73.1-91.8) | 90.3 (80.6-119.3) | 92.5 (84.2-138.1) | 0,378 |
| VEGF | E | pg/ml | 426.8 (235.0-628.2) | 392.1 (242.8-580.5) | 408.1 (210.6-581.6) | 417.5 (221.5-500.5) | 0,622 |
| IGFBP-1 | Me | ng/ml | 4.1 (1.7-7.0) | 4.6 (1.8-6.1) | 4.4 (2.5-6.8) | 5.0 (3.5-7.4) | m: 0,298, f:0,22 |
| IGFBP-2 | Me | ng/ml | 332.6 (254.1-412.5) | 383.7 (260.3-542.4) | 318.6 (236.9-447.8) | 344.7 (292.9-509.7) | 0,406 |
| IGF-I | Me | ng/ml | 148.2 (129.1-163.2) | 134.7 (115.8-147.8) | 145.2 (131.8-164.7) | 127.0 (116.8-144.1) | 0,126 |
| IGF-II | Me | ng/ml | 734.2 (691.8-833.4) | 787.1 (683.8-883.7) | 743.0 (688.6-818.0) | 759.7 (639.0-835.7) | 0,538 |
| Leptin | Me | ng/ml | 3.0 (1.9-5.3) | 4.0 (1.9-8.2) | 3.0 (2.3-6.2) | 4.1 (2.0-9.2) | 0,249 |

Data presented as median (IQR). LME-ANOVA p-value: COPD smokers vs. healthy smokers. M=Method of analysis, TP=total protein, E=ELISA, Me=Mediagnost, EB=Clinical Chemistry, Laboratory EipperBesenthal, Tübingen, Germany
