# Supplementary material for: Repeatability of and Relationship between Potential COPD Biomarkers in Bronchoalveolar Lavage, Bronchial Biopsies, Serum, and Induced Sputum
Source: PLoS One. 2012 Oct 4;7(10):e46207. doi: 10.1371/journal.pone.0046207 (PMC3464239; doi:10.1371/journal.pone.0046207)
Supplement: Table S6 — Markers in urine. (DOC) [file pone.0046207.s008.doc]

Table S6: Markers in urine

| **Analyte** | **M** | **Unit** | **First visit** | | **Second visit** | | **LME-ANOVA** |
| --- | --- | --- | --- | --- | --- | --- | --- |
|  |  |  | **healthy smokers** | **COPD smokers** | **healthy smokers** | **COPD smokers** | **p-value** |
| desmosine(DES) | MS | a.u. | 66.9 (51.3-100.2) | 67.7 (42.1-102.2) | 39.7 (25.2-59.0) | 35.8 (10.1-57.0) | m: 0,89, f:0,059 |
| isodesmosine (iDES) | MS | a.u. | 42.2 (27.9-62.2) | 36.4 (15.7-63.6) | 28.8 (17.3-37.8) | 20.4 (8.0-36.2) | m: 0,444, f:0,021 |
| DES+iDES | MS | a.u. | 9.2 (7.3-13.3) | 8.1 (5.2-11.1) | 10.8 (8.5-14.2) | 8.1 (4.0-12.3) | m: 0,48, f:0,036 |
| DES/Creat. | MS | a.u. | 0.4 (0.4-0.5) | 0.5 (0.4-0.6) | 0.2 (0.2-0.3) | 0.2 (0.2-0.3) | 0,553 |
| iDESCreat. | MS | a.u. | 0.2 (0.2-0.3) | 0.3 (0.2-0.3) | 0.1 (0.1-0.2) | 0.2 (0.1-0.2) | 0,630 |
| DES+iDES/Creat. | MS | a.u. | 0.1 (0.0-0.1) | 0.1 (0.0-0.1) | 0.1 (0.1-0.1) | 0.1 (0.0-0.1) | m: 0,21, f:0,39 |

Data presented as median (IQR). LME-ANOVA p-value: COPD smokers vs. healthy smokers. M=Method of analysis, MS=Mass Spectrometry, a.u.=arbitrary units
