# Supplementary material for: Repeatability of and Relationship between Potential COPD Biomarkers in Bronchoalveolar Lavage, Bronchial Biopsies, Serum, and Induced Sputum
Source: PLoS One. 2012 Oct 4;7(10):e46207. doi: 10.1371/journal.pone.0046207 (PMC3464239; doi:10.1371/journal.pone.0046207)
Supplement: Table S7 — Markers bronchial biopsies. (DOC) [file pone.0046207.s009.doc]

Table S7: Markers bronchial biopsies

| **Analyte** | **M** | **Unit** | **First visit** | | **Second visit** | | **LME-ANOVA** |
| --- | --- | --- | --- | --- | --- | --- | --- |
|  |  |  | **healthy smokers** | **COPD smokers** | **healthy smokers** | **COPD smokers** | **p-value** |
| CD4+LYMPHOCYTES | IC | /0.1MM^2 | 2.9 (1.8-5.4) | 3.0 (1.0-4.5) | 3.2 (2.5-5.4) | 2.7 (1.7-3.9) | 0,152 |
| CD68+CELL | IC | /0.1MM^2 | 1.0 (0.7-1.6) | 1.2 (0.6-1.9) | 1.3 (0.8-1.7) | 1.2 (0.9-1.8) | 0,636 |
| CD8+LYMPHOCYTES | IC | /0.1MM^2 | 3.4 (2.9-5.1) | 3.7 (2.4-4.5) | 4.2 (3.2-5.9) | 4.4 (2.9-5.9) | 0,596 |
| NEUTROPHILS | IC | /0.1MM^2 | 0.6 (0.4-0.8) | 0.6 (0.4-0.8) | 0.7 (0.5-1.1) | 0.6 (0.4-1.0) | 0,697 |

Data presented as median (IQR). LME-ANOVA p-value: COPD smokers vs. healthy smokers. M=Method of analysis,

IC=Immunocytochemistry
