# Supplementary material for: Repeatability of and Relationship between Potential COPD Biomarkers in Bronchoalveolar Lavage, Bronchial Biopsies, Serum, and Induced Sputum
Source: PLoS One. 2012 Oct 4;7(10):e46207. doi: 10.1371/journal.pone.0046207 (PMC3464239; doi:10.1371/journal.pone.0046207)
Supplement: Table S8 — Markers whole blood. (DOC) [file pone.0046207.s010.doc]

Table S8: Markers whole blood

| **Analyte** | **M** | **Unit** | **First visit** | | **Second visit** | | **LME-ANOVA** |
| --- | --- | --- | --- | --- | --- | --- | --- |
|  |  |  | **healthy smokers** | **COPD smokers** | **healthy smokers** | **COPD smokers** | **p-value** |
| LEUKOCYTES | H | G/L | 7.2 (6.0-8.6) | 7.9 (7.5-9.3) | 7.8 (5.8-8.5) | 7.4 (7.0-8.7) | 0,102 |
| NEUTROPHILS | H | % | 59.5 (51.6-63.9) | 58.2 (53.1-63.1) | 58.0 (51.7-62.6) | 58.3 (56.6-64.1) | 0,433 |
| LYMPHOCYTES | H | % | 28.3 (24.5-34.5) | 29.9 (26.3-34.0) | 29.9 (28.1-34.6) | 29.7 (25.0-33.0) | 0,908 |
| MONOCYTES | H | % | 9.1 (7.6-10.0) | 8.4 (7.0-10.2) | 9.0 (7.5-9.8) | 7.2 (6.5-8.2) | 0,766 |
| BASOPHILS | H | % | 0.5 (0.3-0.6) | 0.3 (0.3-0.5) | 0.5 (0.3-0.6) | 0.4 (0.3-0.5) | 0,091 |
| EOSINOPHILS | H | % | 2.6 (1.9-3.4) | 2.2 (1.2-3.2) | 2.7 (1.9-3.6) | 2.1 (1.9-3.0) | 0,281 |
| ERYTHROCYTES | H | T/L | 5.0 (4.7-5.2) | 4.8 (4.7-5.1) | 4.7 (4.6-5.0) | 4.7 (4.4-4.8) | 0,765 |
| HEMATOCRIT | H | % | 44.0 (42.9-47.0) | 45.0 (44.0-46.1) | 43.0 (41.6-44.0) | 44.0 (42.3-45.0) | 0,116 |
| HEMOGLOBIN | H | mM | 9.3 (9.2-9.9) | 9.6 (9.3-9.8) | 9.1 (8.7-9.5) | 9.1 (8.9-9.6) | 0,360 |
| THROMBOCYTES | H | G/L | 242.0 (209.5-278.5) | 246.0 (196.5-290.3) | 238.0 (208.5-273.5) | 242.0 (186.0-300.0) | 0,899 |
| ANISOCYTOSIS | H | % | 44.2 (42.4-46.8) | 46.6 (44.9-47.7) | 45.9 (44.1-46.8) | 46.8 (45.7-47.7) | 0,014 |
| MCH | H | fmol/cell | 1.9 (1.8-1.9) | 1.9 (1.9-1.9) | 1.9 (1.8-1.9) | 1.9 (1.8-1.9) | 0,060 |
| MCHC | H | mM | 21.0 (20.0-21.0) | 20.0 (20.0-21.0) | 21.0 (20.0-21.0) | 20.0 (20.0-21.0) | 0,434 |
| MCV | H | FL | 89.9 (88.2-90.9) | 94.0 (91.2-95.2) | 90.0 (88.6-91.5) | 93.5 (91.0-96.7) | 0,008 |
| INR | Coag |  | 1.0 (0.9-1.0) | 0.9 (0.9-1.0) | 1.0 (0.9-1.0) | 0.9 (0.9-1.0) | 0,071 |
| PTT | Coag | SEC | 32.0 (29.0-32.5) | 31.0 (30.0-32.5) | 32.0 (29.0-34.0) | 32.0 (30.0-33.0) | 0,931 |
| ALKALINE PHOS. | CH | U/L | 73.0 (63.5-88.0) | 78.0 (72.0-104.0) | 74.5 (63.0-84.8) | 89.0 (76.0-96.0) | 0,036 |
| ALT | CH | U/L | 23.0 (18.5-31.5) | 23.5 (21.8-27.8) | 21.0 (17.3-24.8) | 19.0 (15.0-26.0) | 0,554 |
| AST | CH | U/L | 25.0 (22.5-29.5) | 26.0 (23.0-29.0) | 23.0 (20.3-27.0) | 23.0 (22.0-27.0) | 0,347 |
| CREATINE KINASE | CH | U/L | 125.0 (96.5-173.0) | 89.5 (67.8-126.3) | 124.0 (91.0-179.0) | 83.0 (65.0-95.0) | 0,007 |
| CREATININE | CH | µM | 91.0 (82.5-96.5) | 88.0 (79.8-96.0) | 87.5 (83.0-92.3) | 86.0 (78.0-93.0) | 0,585 |
| GAMMA-GT | CH | U/L | 23.0 (21.5-26.5) | 28.0 (24.8-39.3) | 22.0 (19.3-27.3) | 31.0 (22.0-38.0) | 0,086 |
| GLUCOSE | CH | mM | 4.4 (3.9-4.8) | 4.5 (3.7-5.2) | 4.0 (3.5-4.6) | 4.2 (3.8-4.6) | 0,388 |
| POTASSIUM | CH | mM | 4.5 (4.3-5.0) | 4.5 (4.2-4.7) | 4.3 (4.1-4.5) | 4.6 (4.2-4.7) | m: 0,16, f:0,23 |
| SODIUM | CH | mM | 141.0 (139.5-143.0) | 141.5 (139.8-143.0) | 141.0 (139.0-142.0) | 143.0 (139.0-143.0) | 0,751 |
| TOTAL BILIRUBIN | CH | µM | 10.0 (8.0-10.0) | 8.0 (6.0-9.3) | 9.0 (8.0-10.0) | 8.0 (7.0-11.0) | 0,210 |
| pCO2 |  | mmHG | 41.2 (40.2-42.5) | 41.3 (39.3-42.7) | NA | NA | 0,495 |
| pO2 |  | mmHG | 83.7 (75.6-91.3) | 72.3 (69.3-76.1) | NA | NA | 0,000 |

Data presented as median (IQR). LME-ANOVA p-value: COPD smokers vs. healthy smokers. M=Method of analysis, H=haematology, Ch=blood chemistry, Coag=Coagulation
