# Supplementary material for: Repeatability of and Relationship between Potential COPD Biomarkers in Bronchoalveolar Lavage, Bronchial Biopsies, Serum, and Induced Sputum
Source: PLoS One. 2012 Oct 4;7(10):e46207. doi: 10.1371/journal.pone.0046207 (PMC3464239; doi:10.1371/journal.pone.0046207)
Supplement: Text S1 — Methodology of Urine Analysis. (DOC) [file pone.0046207.s011.doc]

**Repeatability of and relationship between potential COPD biomarkers in bronchoalveolar lavage, bronchial biopsies, serum, and induced sputum**

***Manuscript Online Supplement***

### Analysis of Urine samples

Urine (0.5 ml) was mixed with 0.5 ml 0.1 M HCL, centrifuged (5 min, 10g), and applied to a 3 ml MCX cation-exchange column (Oasis, Waters, prewashed with 0.5 ml MeOH, 0.5 ml H2O and 0.5 ml 0.05 M HCL). After washing (2x 1 ml 0.05 M HCL and 4x 1 ml water) and elution (3x 0.1 ml 2M HCL), the eluate was evaporated (40°C under vacuum). The dry residue was dissolved in 1ml 0.1% formic acid and centrifuged. The clear supernatant was used for liquid chromatography-electronspray ionisation-mass spectrometry (LC-ESI-MS) analysis.

### Ionexchange Chromatography:

Column: ProPac SCX-10 2 x 250 mm (Dionex)

Solvents: A: 0.1% Formic Acid, B: 1% NH3 in Water

Flow: 0.5 ml/min, temperature: 40°C

Gradient: t(min) %A %B

0 100 0

1 100 0

5 80 20

7 0 100

7.1 0 100

7.5 100 0

20 100 0

Retention time: Desmosin and Isodesmosin (not separated): 6.2 min

### RP Chromatography:

Column: Atlantis T3 3µm 2.1 x 100 mm (Waters)

Solvents: A: 5 mM ammonia acetate + 7 mM HFBA, B: Methanol / Solvent A 80/20

Flow: 0.5 ml/min, temperature: 50°C

Gradient: t(min) %A %B

0 100 0

1 100 0

9 70 30

10 0 100

11 0 100

11.5 100 0

15 100 0

Retention time: Isodesmosin: 6.2 min

Retention time: Desmosin: 6.7 min
